# Tumor Biomechanics Alters Metastatic Dissemination of Triple Negative Breast Cancer via Rewiring Fatty Acid Metabolism

**DOI:** 10.1002/advs.202307963

**Published:** 2024-04-11

**Authors:** Elysse C. Filipe, Sipiththa Velayuthar, Ashleigh Philp, Max Nobis, Sharissa L. Latham, Amelia L. Parker, Kendelle J. Murphy, Kaitlin Wyllie, Gretel S. Major, Osvaldo Contreras, Ellie T. Y. Mok, Ronaldo F. Enriquez, Suzanne McGowan, Kristen Feher, Lake‐Ee Quek, Sarah E. Hancock, Michelle Yam, Emmi Tran, Yordanos F. I. Setargew, Joanna N. Skhinas, Jessica L. Chitty, Monica Phimmachanh, Jeremy Z. R. Han, Antonia L. Cadell, Michael Papanicolaou, Hadi Mahmodi, Beata Kiedik, Simon Junankar, Samuel E. Ross, Natasha Lam, Rhiannon Coulson, Jessica Yang, Anaiis Zaratzian, Andrew M. Da Silva, Michael Tayao, Ian L. Chin, Aurélie Cazet, Maya Kansara, Davendra Segara, Andrew Parker, Andrew J. Hoy, Richard P. Harvey, Ozren Bogdanovic, Paul Timpson, David R. Croucher, Elgene Lim, Alexander Swarbrick, Jeff Holst, Nigel Turner, Yu Suk Choi, Irina V. Kabakova, Andrew Philp, Thomas R. Cox

**Affiliations:** ^1^ Cancer Ecosystems Program Garvan Institute of Medical Research and The Kinghorn Cancer Centre Darlinghurst NSW 2010 Australia; ^2^ School of Clinical Medicine St Vincent's Clinical Campus UNSW Medicine & Health UNSW Sydney Sydney 2010 Australia; ^3^ Centenary Institute Camperdown NSW 2050 Australia; ^4^ Present address: Intravital Imaging Expertise Center VIB Center for Cancer Biology, VIB Leuven 3000 Belgium; ^5^ Victor Chang Cardiac Research Institute Darlinghurst NSW 2010 Australia; ^6^ South Australian immunoGENomics Cancer Institute (SAiGENCI) Adelaide SA 5005 Australia; ^7^ School of Mathematics and Statistics, Charles Perkins Centre University of Sydney Sydney 2050 Australia; ^8^ School of Biomedical Sciences UNSW Sydney Sydney 2033 Australia; ^9^ School of Life Sciences University of Technology Sydney Sydney NSW 2007 Australia; ^10^ School of Mathematical and Physical Sciences University of Technology Sydney Sydney NSW 2007 Australia; ^11^ School of Human Sciences University of Western Australia Crawley WA 6009 Australia; ^12^ St Vincent's Hospital Darlinghurst NSW 2010 Australia; ^13^ Department of Pathology St. Vincent's Hospital Sydney 2010 Australia; ^14^ School of Medical Sciences Charles Perkins Centre Faculty of Medicine and Health University of Sydney Sydney NSW 2050 Australia; ^15^ School of Biotechnology and Biomolecular Sciences UNSW Sydney Sydney 2033 Australia; ^16^ Biology of Ageing Laboratory and Centre for Healthy Ageing Centenary Institute Missenden Road, Camperdown Sydney NSW 2050 Australia; ^17^ School of Sport Exercise and Rehabilitation Sciences University of Technology Sydney Ultimo NSW 2007 Australia

**Keywords:** biomechanics, breast cancer, extracellular matrix, metabolism, metastasis

## Abstract

In recent decades, the role of tumor biomechanics on cancer cell behavior at the primary site has been increasingly appreciated. However, the effect of primary tumor biomechanics on the latter stages of the metastatic cascade, such as metastatic seeding of secondary sites and outgrowth remains underappreciated. This work sought to address this in the context of triple negative breast cancer (TNBC), a cancer type known to aggressively disseminate at all stages of disease progression. Using mechanically tuneable model systems, mimicking the range of stiffness's typically found within breast tumors, it is found that, contrary to expectations, cancer cells exposed to softer microenvironments are more able to colonize secondary tissues. It is shown that heightened cell survival is driven by enhanced metabolism of fatty acids within TNBC cells exposed to softer microenvironments. It is demonstrated that uncoupling cellular mechanosensing through integrin β1 blocking antibody effectively causes stiff primed TNBC cells to behave like their soft counterparts, both in vitro and in vivo. This work is the first to show that softer tumor microenvironments may be contributing to changes in disease outcome by imprinting on TNBC cells a greater metabolic flexibility and conferring discrete cell survival advantages.

## Graphical abstract

Triple Negative Breast Cancer cells primed in biomechanically soft or stiff microenvironments demonstrate striking differences in cell behavior, which has important consequences for the different aspects of metastatic dissemination. Biomechanical priming in stiff microenvironments drives enhanced cell proliferation and three‐dimensional invasion, whereas biomechanical priming in softer microenvironments increases anchorage independent survival, resistance to shear stress, and contrary to expectation, increases cell survival and outgrowth at secondary organs. Changes in cellular metabolism (fatty acid oxidation in particular) appear to underpin these striking differences.

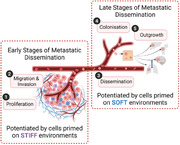

## 1 Introduction

The extracellular matrix (ECM) is known to be highly dysregulated in cancer, with altered expression, deposition, and organization of a range of ECM components that together, alter the behavior of both cancer and stromal cells and contribute to disease progression.^[^ ^1^ ^]^ Beyond the biochemical effects that these ECM changes elicit in cells, there are also significant biophysical effects of the tumor ECM that alter cellular behavior within the primary tumor. Many solid tumors exhibit significant changes in local forces and tissue viscoelastic properties both spatially, within a single tumor, and over time throughout disease progression.^[^ ^2^, ^3^ ^]^ Indeed, it is already known that an increase in mammary tissue stiffness accompanies breast tumor formation and progression,^[^ ^4^, ^5^ ^]^ largely due to the increased production and deposition of extracellular matrix components.^[^ ^1^ ^]^ Research in the field has also elucidated significant, biologically relevant effects of changes in biophysical properties, from seminal work demonstrating the influence of stiffness on the malignant phenotype of cells,^[^ ^6^, ^7^ ^]^ to the regulation of epithelial to mesenchymal transition,^[^ ^8^, ^9^ ^]^ enhanced tumor cell invasiveness,^[^ ^4^, ^10^, ^11^ ^]^ and resistance to chemotherapies.^[^ ^9^, ^12^ ^]^ However, most of the data thus far relates to the effect of stiffness on cells within the primary tumor, with research still lacking into the longer‐term effects of primary tumor biomechanics on cancer cells once they have left the primary tumor, including during metastatic dissemination, survival, and overt colonization of secondary sites.

In recent years, the role of cellular energetics throughout tumor progression has become increasingly appreciated. Indeed, it is known that cancer cells adjust their metabolic profile to best adapt to each unique phase of the metastatic cascade.^[^ ^13^, ^14^, ^15^, ^16^ ^]^ In triple negative breast cancer (TNBC) specifically, it is known that while there is variability in the dominating metabolic phenotype of cancer cells within the primary tumor, the prevalence of glycolytic dependency is higher among the triple negative subtype group when compared to other subtypes of breast cancer.^[^ ^17^, ^18^, ^19^, ^20^, ^21^ ^]^ As with other tumor types, glycolysis is often adopted at the proliferative stages of tumor growth, where glycolytic intermediates aid in the production of essential building blocks for cell proliferation (i.e., lipids, nucleotides and amino‐acids), thus fueling the increase in biomass characteristic of, and indeed necessary for cancer progression.^[^ ^13^, ^15^, ^16^ ^]^ Of particular interest though, are the metabolic changes that are thought to occur during the processes of metastatic dissemination, secondary site engraftment, and survival.^[^ ^13^, ^22^, ^23^, ^24^ ^]^ Here, the metabolic flexibility characteristic of TNBC^[^ ^17^, ^25^ ^]^ enables cells to adapt their cellular metabolism. Indeed, recent work has shown that TNBC metastatic lesions alter their metabolic profiles the better adapt to their destination tissues, with robust enrichment of metabolic pathways (oxidative phosphorylation and fatty acid metabolism), particularly prevalent within lung and liver lesions.^[^ ^25^, ^26^ ^]^ These metabolic shifts have been shown to promote cell survival by enabling disseminated cancer cells to utilize alternative fuel sources (in nutrient‐poor environments in particular) while also producing oxidative stress detoxifying co‐factors such as nicotinamide adenine dinucleotide phosphate (NADPH).^[^ ^27^, ^28^ ^]^ This idea of metabolic flexibility is thought to provide a critical advantage in the stages of cancer cell dissemination and survival in TNBC, and even other cancer types more broadly,^[^ ^24^, ^29^ ^]^ However, the underlying cues that trigger and guide this adaptation are still under intense investigation.

Using experimental models of metastatic dissemination, survival, and outgrowth, we show that the biomechanical properties of the primary tumor microenvironment critically influence the metastatic capacity of TNBC cells. Interestingly, we found that softer tumor microenvironments primed cancer cells with enhanced survival mechanisms both in vitro and in vivo that consequently increases metastatic colonization of secondary sites. Further, we found this to be intimately linked to the metabolic profile of cells primed in soft or stiff microenvironments (recapitulating the differences in healthy and tumor tissue, as well as heterogeneity within tumors) with biomechanically induced re‐wiring of fatty acid oxidation. Subsequently, upon leaving the primary tumor, this altered metabolic profile renders them more able to colonize the secondary metastatic niche. Finally, we demonstrate that the priming effects of soft microenvironments can be recapitulated in cancer cells in stiff microenvironments through blocking β1‐integrin mediated mechanosensing, which leads to an increase in fatty acid metabolism and subsequent increase in survival in in vitro and in vivo models of metastatic colonization.

## 2 Results

### 2.1 Modelling Biomechanical Tumor Heterogeneity in vitro

**Figure 1:**
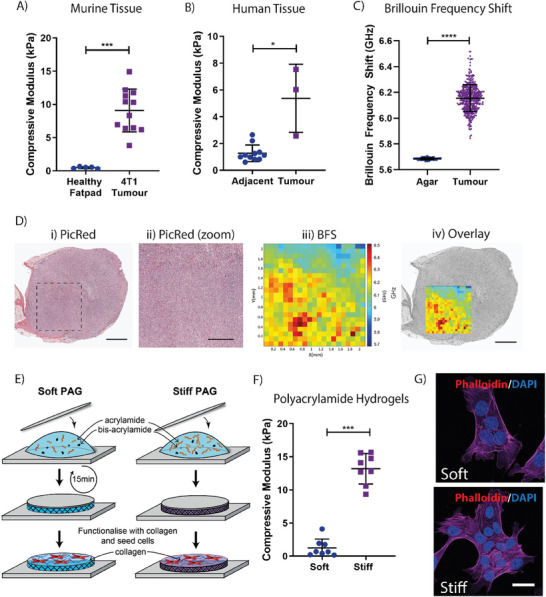
Mimicking the biomechanical properties of healthy and tumor tissues in vitro. A) Biomechanical profiling of healthy murine fat pads and 4T1 murine mammary carcinoma cell generated orthotopic tumors. *n* = 5–12. B) Biomechanical profiling of human tumor tissues, alongside adjacent, healthy tissue. *n* = 3 tumor tissues, with 3–4 matched healthy tissues per patient. C) Brillouin Frequency Shift within a single 2mm^2^ tumor section, when compared to the control material, agar. D) i) Histological staining of mouse mammary tumor used for Brillouin Microscopy (scale bar = 1 mm); ii) zoom in of the region measured by Brillouin Microscopy (scale bar = 500 µm); iii) 2D mapping of the Brillouin frequency shift across the surface of the tumor and iv) overlay of the mapped region onto the histological cross section of the tumor (scale bar = 1 mm). Images representative of 1 tumor sample E) Schematic of polyacrylamide hydrogel pipeline, depicting the generation of soft and stiff hydrogels. F) Biomechanical profiling of the polyacrylamide hydrogels using unconfined compression. *n* = 8. G) Immunocytochemical staining of the actin cytoskeleton in 4T1 murine mammary carcinoma cells with Phalloidin (Magenta) and 4',6‐diamidoino‐2‐phenylindole (DAPI; Blue) seeded onto soft and stiff hydrogels. Scale bar = 50 µm. Statistical testing throughout performed using the Mann‐Whitney U test, * = *p* < 0.05, *** = *p*<0.001, **** = *p* < 0.0001.

The biomechanical properties of solid tumors are diverse and vary dynamically throughout disease progression^[^ ^2^, ^4^ ^]^; between tumor sub‐types^[^ ^30^, ^31^, ^32^ ^]^; and often within a single tumor.^[^ ^33^, ^34^ ^]^ Changes in tissue stiffness are known to feed into tumor progression, yet little is known about how this biomechanical heterogeneity contributes to tumor heterogeneity. Biomechanical characterization of *ex vivo* tissue from murine 4T1 orthotopic mammary tumors and aged‐matched healthy mammary fat pads confirmed an 18.7‐fold difference between the healthy and tumor tissues (0.49 ± 1.4 kPa and 9.09 ± 3.2 kPa, respectively (Mean ± SD, **Figure** **1**A)). Similarly, bulk unconfined compression analysis of healthy human breast tissue and adjacent triple‐negative breast tumor tissue taken from patients undergoing resection also confirmed elevated tumor stiffness (adjacent 1.27 ± 0.6 kPa and tumor 5.38 ± 2.5 kPa respectively (Mean ± SD, Figure 1B)).

While increases in bulk tissue stiffness are well acknowledged to occur during breast tumor development,^[^ ^35^ ^]^ intra‐tumor biomechanical heterogeneity is less well studied. To assess the biomechanical heterogeneity within a single tumor, Brillouin microscopy was applied across a whole bisected 4T1 mammary tumor.^[^ ^36^ ^]^ Brillouin microscopy is a label‐free and non‐destructive optical elastography‐based technique that measures the viscoelastic properties of biological samples.^[^ ^37^, ^38^ ^]^ We observed a significant 0.468 GHz variation in Brillouin frequency shift (BFS) across the tumor (Figure 1C), a variation 10‐fold greater than the supporting agar (which is homogeneous in nature) in which the tumor was embedded. This BFS variation results from differences in local microscopic elasticity of the tumors.^[^ ^37^ ^]^ Furthermore, the linewidth (LW) of Brillouin peaks, which is a measure of local heterogeneity within the 3D voxel (100 µm^3^) at each sampling coordinate,^[^ ^37^, ^38^ ^]^ also exhibited significant variability (Figure S1A,B, Supporting Information). Co‐registration of the BFS map with histology indicates higher BFS toward the periphery of the tumor, with lower BFS co‐localizing with the core of the tumor. These results are in line with previously published data from other methodologies which have shown ECM dense regions toward the periphery of the tumor resulting in regions of increased stiffness^[^ ^33^ ^]^ (Figure 1D). Furthermore, this is the first time Brillouin microscopy has been deployed in the solid tumor setting demonstrating proof‐of‐principle application for future studies.

To mimic this heterogeneity in biomechanical properties, we used collagen I functionalized polyacrylamide hydrogels (PAGs, Figure 1E) with biomechanical properties tailored to reflect the stiffness of healthy and tumor tissue. Bulk, unconfined compression analysis confirmed the stiffnesses of 1.24 ± 1.3 kPa and 13.20 ± 2.3 kPa, respectively, herein named “Soft” and “Stiff” (Mean ± SD, Figure 1F). Orthogonal biomechanical characterization of the soft and stiff polyacrylamide hydrogels using atomic force microscopy (AFM) force mapping confirmed moduli of 1.31 ± 0.3 kPa (soft) versus 8.98 ± 1.8 kPa (stiff) (Mean ± SD). Shear rheology (Soft: 190.6 ± 32 G’; Stiff: 1503 ± 255 G’; Mean ± SD) showed similar trends (Figure S1C,D, Supporting Information). Finally, measurement of the microscopic viscoelastic properties of the hydrogels using Brillouin microscopy^[^ ^37^, ^38^ ^]^ similarly showed a significant increase in Brillouin frequency in stiffer hydrogels when compared to the softer hydrogels, as seen in primary tumor samples (Figure S1E, Supporting Information).

To confirm the effect of substrate stiffness on breast cancer cell behavior, the 4T1 murine mammary carcinoma cell line was then seeded directly onto collagen functionalized soft and stiff polyacrylamide hydrogels. 4T1 cells readily adhered to and spread on the surface of both the soft and stiff polyacrylamide hydrogels with no discernible phenotypic difference (Figure 1G). These data confirm that this approach would be an excellent model to dissect intracellular effects of biomechanical priming.

### 2.2 Biomechanical Priming Affects Multiple Elements of Breast Cancer Cell Behavior in vitro

**Figure 2:**
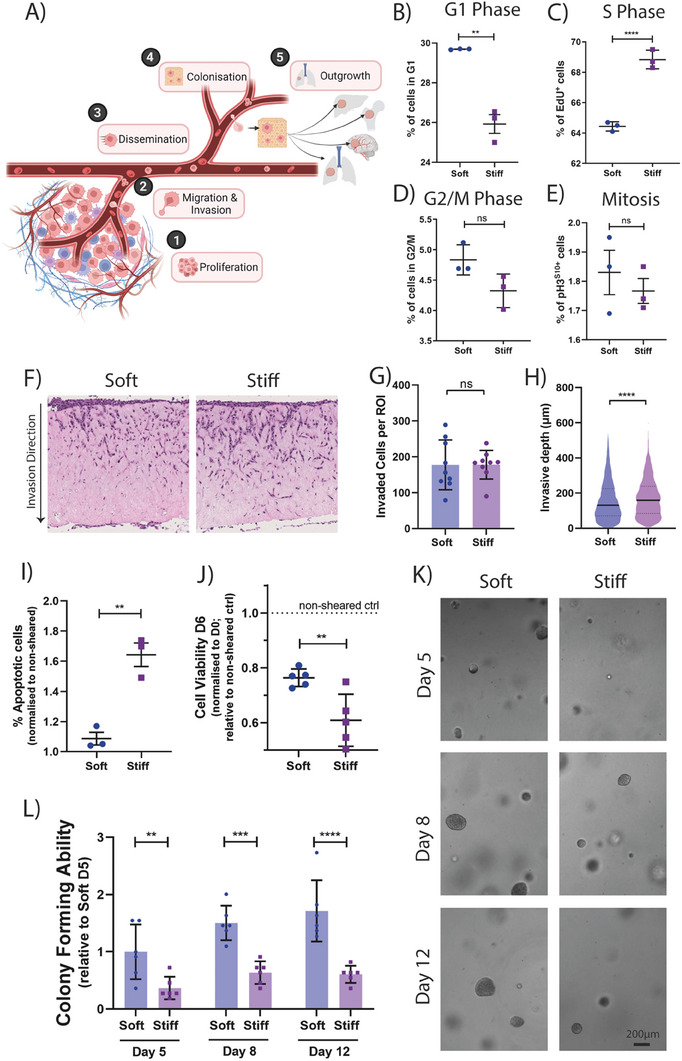
Microenvironmental stiffness affects mammary carcinoma cell behavior in vitro A) Schematic of the metastatic cascade, depicting the 5 main stages of disease progression. Created with BioRender.com. B) Respective quantifications of the proportion of cells in G1, C) S Phase, D) G2/M, and E) Mitosis as measured by EdU incorporation after a single 1h pulse with EdU monomer or proportion of pHistone3 positive cells while on soft or stiff conditions. *n* = 3 biological repeats. F) Representative bright field image of stiffness conditioned cells invading into an organotypic collagen plug. Respective quantification of G) Number of invaded cells per region of interest (ROI) and H) Invasive depth of cells. Graphs depict one biological repeat, representative of *n* = 4 biological repeats. I) Proportion of apoptotic cells post shearing after conditioning on soft and stiff, normalized to the non‐sheared controls. *n* = 3 biological repeats. J) Cell viability of sheared cells at 6 days post shearing, normalized to day 0 cell number and relative to non‐sheared controls. Graph depicts one biological repeat, representative of *n* = 3 biological repeats. K) Representative bright field images of stiffness preconditioned 4T1 cells embedded in a 3D matrix on day 5, 8, and 12 of culture. Scale bar = 500 µm. L) Relative quantification of the spheroid forming ability of stiffness preconditioned 4T1 cells. Graph depicts one biological repeat, representative of *n* = 4 biological repeats. Statistical testing performed using Mann‐Whitney U. Statistical testing performed using two‐sided unpaired *t*‐tests throughout, unless specified otherwise ** = *p* < 0.01, *** = *p* < 0.001, **** = *p* < 0.0001.

Tumor progression is a multi‐step process (**Figure** **2**A), with cancer initiating at the proliferative, primary tumor stage, followed by progression through the metastatic cascade. We therefore sought to determine the effects of biomechanical priming on these various stages of disease. To assess proliferation at the primary site (Figure 2A; Stage 1), we assessed cell cycle distribution using EdU pulse incorporation flow cytometry and staining for phospho‐Histone H3 (ser10) (Figure S2A,B, Supporting Information). In keeping with other studies, we confirmed that soft substrates lead to an increase in cells in G1 (Figure 2B) and decrease in cells in S‐phase of the cell cycle (Figure 2C), compared to cells cultured on stiff substrates. There was no significant change in the number of cells in G2‐M phase between these two conditions, nor in the proportion of phosphorylated Histone H3 (mitotic cells; Figure 2D,E). These results confirm that cancer cells in stiffer microenvironments progress through the G1‐S phases of the cell cycle faster, indicative of enhanced proliferation of stiff cultured cells.^[^ ^39^ ^]^

To determine whether biomechanical priming selectively enriches for sub‐populations from within the global population, we performed a time course analysis using DNA barcoded 4T1 cells^[^ ^40^, ^41^ ^]^ cultured on soft and stiff microenvironments for up to 6 days. Genetic sequencing of the genetic barcode pool at 24, 72, and 144 h confirmed no significant changes in barcode diversity as a function of biomechanical stiffness (Figure S2C, Supporting Information). A loss in barcode diversity would indicate clonal enrichment, however our results suggest that the biomechanical properties of the microenvironment are not leading to selection of more aggressive clones already present within the population.

Next, to mimic the initial stages of cell invasion away from the primary tumor (Figure 2A; Stage 2) we assessed the invasive capacity of soft and stiff primed cells through a 3D physiologically relevant, collagen‐rich matrix. Here, biomechanically primed cancer cells are seeded onto organotypic plugs and allowed to invade (Figure 2F). Our data show that while there was no difference in the total number of invaded cells between the soft and stiff condition (Figure 2G), the stiff primed cells invaded slightly deeper into the collagen plugs (157.3 µm vs 172.0 µm; soft vs stiff respectively; Figure 2H). These data confirm a moderately enhanced invasive capacity of the stiff primed cells away from the primary tumor.

It has been previously established that the deformability of cells is linked to their invasive potential, with more deformable cancer cells being linked to increased invasion.^[^ ^42^, ^43^ ^]^ To assess biomechanical properties at the single cell level, we performed both single cell AFM,^[^ ^44^ ^]^ and real‐time deformability cytometry (RT‐DC)^[^ ^45^ ^]^ studies on soft and stiff primed cells. Both methodologies robustly showed a significant decrease in the stiffness (Figure S2D, Supporting Information) and increase in the deformability of stiff primed cells (Figure S2E–G, Supporting Information), compared to soft. These data together suggest that while both soft and stiff primed cells invaded into the collagen matrices, the enhanced invasive depth achieved by the stiff primed cells may in part be due to their increased deformability, allowing cells to better navigate the fibrous matrices of the organotypic plugs. Importantly, our data also confirm that the changes in intracellular stiffness induced by the biomechanical properties of the substrate (AFM data) are maintained when cells detach and enter into the RT‐DC microfluidics, suggesting that biomechanical priming would persist when metastasising cancer cells enter the circulation.

During circulation in the blood, cancer cells are exposed to large shear stresses that cause cellular damage through a range of mechanisms that subsequently impact a cancer cell's metastatic potential.^[^ ^46^ ^]^ Of note, stiffer, less deformable cells are often associated with increased resistance to shear stress.^[^ ^47^ ^]^ To assess this (Figure 2A; Stage 3), we subjected soft and stiff primed cells to shear stress (mimicking forces experienced in the circulation) before assessing shear induced apoptosis by flow cytometry. We found that breast cancer cells primed on soft matrices (which generates less deformable cells) exhibit increased resistance to shear stresses, leading to higher viability post shear at 24h (Figure 2I) and 6 days (Figure 2J). These data support the notion that the changes in intracellular stiffness induced by the biomechanical properties of the primary tumor are also maintained when cells detach from the primary tumor and enter into the circulation.

Finally, to understand the effect of biomechanical priming on metastatic colonization and outgrowth at secondary sites (Figure 2A; Stages 4 & 5), we assessed the in vitro 3D spheroid forming ability of biomechanically primed cancer cells. 4T1 cells were primed (soft versus stiff) followed by embedding as a single cell suspension into a 3D alginate‐Collagen I interpenetrating network hydrogel. Our results demonstrate that soft primed cells begin forming spheroids faster, with 2.1‐fold more spheroids at day 5 compared to stiff (Figure 2K,L). Measurements at day 8 and 12 showed a similar 1.8‐fold increase in spheroid number. These data indicate that cancer cells biomechanically primed by softer microenvironments exhibit greater spheroid forming capacity when seeded as single cells within a new environment. To confirm that our observations were not a consequence of changes in overall cell viability upon seeding, we performed a propidium iodide (PI)/Hoechst staining and found no significant difference between conditions (Figure S2H, Supporting Information), confirming that stiff conditioned cells remained as viable, but indolent single cells within the matrices, while the soft primed cells rapidly begin proliferating.

Overall, our data demonstrate the metastatic stage specific effects of biomechanics on cancer cell behavior, with both soft and stiff primed cells being endowed with discrete, but unique advantages pertinent to different points of the metastatic cascade.

### 2.3 Biomechanical Priming Alters Efficiency of Metastatic Dissemination in vivo

**Figure 3:**
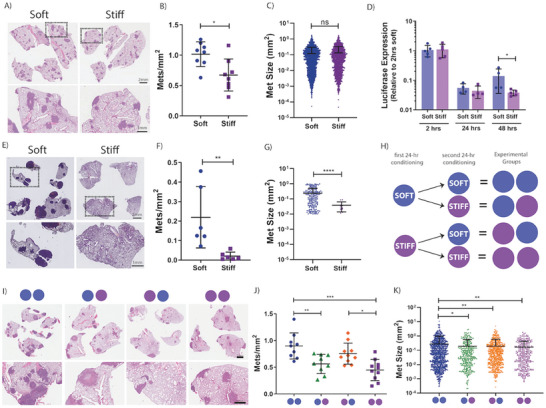
Biomechanical properties of the microenvironment affect metastatic capacity in vivo. A) Representative hematoxylin & eosin (H&E) image of murine lungs, 3 weeks after intravenous injection of stiffness preconditioned 4T1 mammary carcinoma cells. B) Quantification of the number of metastatic lesions/mm^2^ of lung tissue. Quantification from 3 stepped sections per mouse, *n* = 8 mice per group. C) Distribution plot of metastatic lesion size. D) Relative cancer cell abundance in the lungs of mice at 2 h, 24 h, and 48 h post intravenous injection of stiffness preconditioned 4T1 mammary carcinoma cells, as measured by multiplex quantitative polymerase chain reaction (qPCR). *n* = 4–5 mice per group, per time point. E) Representative H&E image of murine lungs, 3 weeks after intravenous injection of stiffness preconditioned E0771 cells. F) Quantification of the number of metastatic lesions/mm^2^ of lung tissue. Quantification from 3 stepped sections per mouse, *n* = 6 mice per group. G) Distribution plot of metastatic lesion size. H) Visual representation of the experimental design from our flip‐stiffness study. I) Representative H&E image of murine lungs, 3 weeks after intravenous injection of stiffness preconditioned 4T1 mammary carcinoma cells, as per experimental design in H). J) Quantification of the number of metastatic lesions/mm^2^ of lung tissue. Statistical testing performed using a one‐way ANOVA. Quantification from 3 stepped sections per mouse, *n* = 9‐10 mice per group. K) Distribution plot of metastatic lesion size. Statistical testing performed using a one‐way ANOVA. Statistical testing performed using the Mann‐Whitney U test throughout, unless indicated otherwise * = *p*<0.05, ** = *p* < 0.01, *** = *p* < 0.001, **** = *p* < 0.0001.

To confirm our findings that soft‐conditioned cells have enhanced capacity for colonization and outgrowth at secondary sites, we used the experimental (tail vein) model of lung metastasis. Briefly, biomechanically primed 4T1 mammary carcinoma cells (soft vs stiff) were injected into the tail vein of mice, and metastatic colonization of the lung determined at 21 days by histological analysis.^[^ ^48^ ^]^ Supporting our in vitro data, we observed a significant 1.5 ± 0.3‐fold increase in the number of metastatic lesions formed within the lungs of mice injected with soft primed 4T1 cells compared to stiff primed (**Figure** **3**A,B). However, there was no significant difference in the average size of the metastatic lesions (Figure 3C).

Next, to assess cancer cell colonization dynamics during the early stages of cell seeding, we tail vein injected a separate cohort of mice and lungs were harvested at early time points of 2‐, 24‐, and 48‐hours post injection. Cancer cell burden within the lungs at these early time points was quantified using multiplex qPCR on lung tissue for vimentin (present in all cells) and luciferase (present in cancer cells only). Data showed no significant difference between the cancer cell burden of soft and stiff primed cells arriving and/or present in the lungs at 2‐ or 24‐hours post injection. However, at 48 h post injection, cancer cell presence began to significantly increase in the soft biomechanically primed condition compared to stiff (Figure 3D). These data indicate that the soft primed cells are more readily able to activate proliferative pathways and progress from single cells to micro metastatic lesions, data which is consistent with the results obtained from our in vitro spheroid forming assays.

This enhanced in vivo pulmonary colonizing capacity of soft conditioned cells was confirmed with a second triple‐negative breast cancer cell line, the E0771 syngeneic model. Confirming the data obtained in the 4T1 model, E0771 breast cancer cells also showed a significant increase in the number of metastatic lesions and overall metastatic burden when primed on soft microenvironments prior to tail vein injection (Figure 3E,F; Figure S3A, Supporting Information). Furthermore, this cell line also displayed a significant increase in metastatic lesion size in the soft primed condition compared to stiff primed cells (Figure 3G).

Given the biomechanical heterogeneity of the tumor microenvironment, cancer cells will encounter varying stiffnesses over both time and space. Considering our data demonstrating that biomechanical conditioning did not support clonal selection (Figure S2C, Supporting Information), we sought to investigate the longevity of this biomechanically enhanced in vivo colonization phenotype. Previous evidence in normal fibroblasts has suggested that cells retain information about their previous biomechanical cues^[^ ^49^ ^]^ with experiments showing that normal cells mechanically primed on stiff substrates retain their “stiff” phenotype when transferred to soft matrices and vice versa. Since this seminal work, biomechanical memory has been demonstrated to occur in bone marrow stromal cells,^[^ ^8^, ^50^ ^]^ myofibroblasts,^[^ ^51^ ^]^ and epithelial cells.^[^ ^52^, ^53^ ^]^ However, to date, there is limited investigation into the role for this effect in cancer cells in solid tumors. To assess this, prior to tail vein injection, 4T1 mammary carcinoma cells were primed on soft or stiff substrates for 24 hours, followed by an additional biomechanical priming period on the opposite (or same) stiffness, for a further 24‐hour period (Figure 3H). Breast cancer cells primed twice on soft microenvironments (2 × 24 h) were the most aggressive, leading to the greatest metastatic pulmonary burden compared to all other conditions, Of note is that soft conditioned cells (24 h) subjected to a second conditioning period on stiff (24 h), still exhibited an increase in the number of metastatic lesions/mm^2^ when compared to the 2 × 24 h stiff cohort, supporting the existence of a longer term effect of the biomechanical priming of the cells during the initial 24 hours on soft substrates which is not fully reversed by exposure to the stiffer substrates (Figure 3I**–**K; Figure S3B–D, Supporting Information).

### 2.4 Biomechanical Priming Alters Intracellular Glucose Dynamics and Mitochondrial Respiration

During our initial experiments assessing cell behavior in vitro, we observed a small, but consistently higher signal at day 0 in soft primed cells (despite seeding equal cell numbers) as measured by AlamarBlue (Figure S4A, Supporting Information). AlamarBlue is a resazurin‐based assay that is an important redox indicator used to evaluate metabolic function and cellular health.^[^ ^54^ ^]^ In light of this, we hypothesized that the priming of cells on soft or stiff microenvironments may be affecting basal cell metabolism, which could then be subsequently affecting the observed metastatic propensity of cells, both in vitro and in vivo. Indeed, in recent years there have been several reports confirming that tissue biomechanics alter mitochondrial function, structure, and activity with downstream effects on phenotype, such as altered cell contractility and survival.^[^ ^55^, ^56^, ^57^, ^58^, ^59^ ^]^ More specifically, studies have found that softer microenvironments enhance lipid centric metabolic processes such as fatty acid synthesis, accumulation, and oxidation.^[^ ^60^, ^61^ ^]^ Alteration of cellular metabolic processes has recently emerged as an important hallmark of cancer,^[^ ^62^ ^]^ with numerous studies implicating altered metabolic processes in the development and progression of cancer^[^ ^63^, ^64^ ^]^ and a specific emphasis on the metabolic alterations within circulating tumor cells.^[^ ^24^, ^65^ ^]^

**Figure 4:**
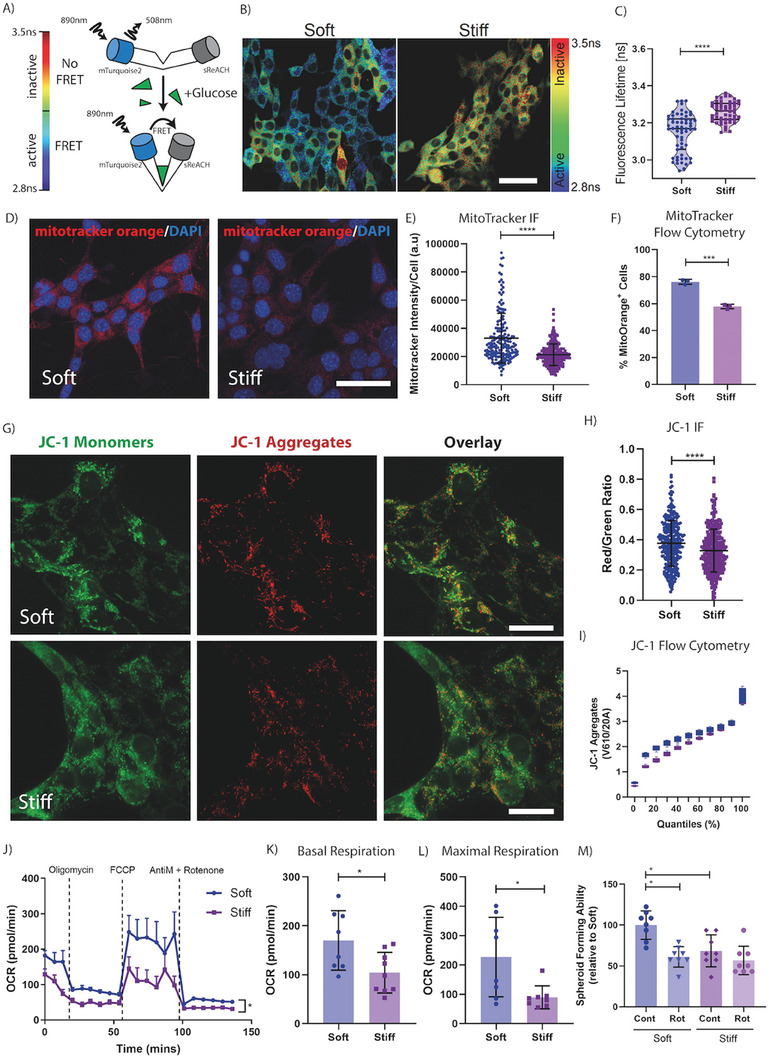
Biomechanical properties of the microenvironment affect mitochondrial respiration. A) Schematic of the glucose biosensor transfected into our 4T1 cell line, representing the active and inactive conformations and their respective heatmap ranges, with blue/green cells indicating high glucose uptake and yellow/red indicative of low glucose levels. B) Representative fluorescence lifetime imaging (FLIM)‐FRET image of our 4T1 cells cultured directly on the soft and stiff microenvironments. Scale bar = 50 µm. C) Quantification of the fluorescence lifetime of each cell on the soft or stiff microenvironments. Graph depicts one biological repeat, representative of *n* = 3 biological repeats. D) Representative fluorescence image of 4T1 cells, stained with MitoTracker orange, on soft and stiff microenvironments. Scale bar = 50 µm. E) Quantification of MitoTracker orange intensity/cell from the immunofluorescent images. Graph depicts one biological repeat, representative of *n* = 2 biological repeats. F) Quantification of MitoTracker positivity as measured by flow cytometry. Graph depicts one biological repeat, representative of *n* = 2 biological repeats. Statistical testing performed using a two‐sided unpaired *t*‐test with Welches correction. G) Representative images of 4T1 cells on soft or stiff microenvironments, stained with mitochondrial dye JC‐1. Imaging of JC‐1 monomers (Green), aggregates (Red) and overlayed. Scale bar = 25 µm. H) Quantification of the red/green JC‐1 ratio per cell from the immunofluorescence images. Graph depicts one biological repeat, representative of *n* = 2 biological repeats. I) Quantification of JC‐1 aggregate^+^ population as measured by flow cytometry with cells binned into quantiles based on the JC‐1 aggregate levels. Graph depicts one biological repeat, representative of *n* = 2 biological repeats. Statistical testing performed using the Kolmogorov‐Smirnov test, with p < 1E‐10. J) Seahorse bioanalyzer plot for 4T1 mammary carcinoma cells, preconditioned on soft or stiff microenvironments with equal numbers embedded in a 3D alginate hydrogel bead. Statistical testing performed with a two‐way ANOVA. Including quantification of K) Basal respiration and L) Maximal respiration values. M) Relative quantification of spheroid forming ability of stiffness preconditioned 4T1 cells, with and without oxidative phosphorylation inhibitor Rotenone (Rot). Graph depicts one biological repeat, representative of *n* = 3 biological repeats. Statistical testing performed using the Mann‐Whitney U test throughout, unless indicated otherwise * = *p* < 0.05, *** = *p* < 0.001, **** = *p* < 0.0001.

Considering these recent works, we sought to assess whether the mechanical priming of cells was affecting the metastatic propensity of cells via alterations in cellular energetics. First, we employed a glucose Förster resonance energy transfer (FRET) biosensor, which uses the bacterial glucose transporter MglB^[^ ^66^ ^]^ to track and quantify intracellular levels of glucose (**Figure** **4**A) by measuring fluorescence lifetime of the donor fluorophore mTurquoise2 (a measure of MglB‐glucose interaction). We observed an increase in intracellular glucose in soft primed cells, evidenced by a decrease in mTurquoise2 fluorescence lifetime (Figure 4B). We also observed an increased range of the fluorescence lifetime of mTurquoise2 in soft primed cells indicating a greater variability of intracellular glucose levels (Figure 4B,C).

Further complementary interrogation was then carried out using a suite of metabolic dyes and assays to assess mitochondrial health and activity. Mitotracker orange and JC‐1 are frequently used to monitor mitochondrial membrane potential, a surrogate for mitochondrial health^[^ ^67^, ^68^ ^]^ and mitochondrial activity.^[^ ^69^, ^70^, ^71^, ^72^ ^]^ Using the mitotracker orange in both imaging and flow cytometry applications, we observed an increase in mitotracker orange signal per cell in soft primed cells compared to stiff (Figure 4D**–**F; Figure S4B, Supporting Information). Supporting these data, we also used the JC‐1 dye, which shifts its fluorescent profile from green to red when taken up by mitochondria with a high mitochondrial membrane potential. We observed a similar increase in the red/green ratio in soft primed cells, matching the mitotracker orange data. Both these data confirm an increased mitochondrial health and activity in the soft primed cells compared to stiff (Figure 4G**–**I; Figure S4C,D, Supporting Information).

Mitotracker orange and JC‐1 are indirect indicators of altered mitochondrial activity and health in response to biomechanical priming. To confirm these findings, we next measured mitochondrial respiration directly using a Seahorse XFe24 bioanalyzer which measures oxygen consumption rates in real‐time.^[^ ^73^, ^74^ ^]^ We observed a significant increase in basal respiration (Figure 4J,K) and maximal respiration (Figure 4L) as well as proton leak, adenosine triphosphate (ATP) synthesis and spare capacity (Figure S4E–G, Supporting Information) in soft versus stiff biomechanically primed cancer cells. These data together indicate an increase in oxidative metabolism in soft primed cells and confirm that biomechanical priming of mammary carcinoma cells in soft microenvironments can trigger a significant shift in cellular energetics.

To determine whether the shift toward mitochondrial metabolism in soft primed cells was playing a role in the spheroid forming capacity observed in Figure 2, we repeated the spheroid forming assays in the presence of rotenone, an inhibitor of complex I of the electron transport chain which limits mitochondrial oxidative phosphorylation. Our data confirmed a significant sensitivity of soft primed cells to rotenone (Rot) in terms of spheroid forming ability, while stiff primed cells remained relatively unaffected (Figure 4M). These results confirm that the increased reliance on mitochondrial metabolism in soft primed cancer cells likely underpins their observed increased spheroid forming capacity.

### 2.5 Biomechanical Conditioning Leads to Altered Cellular Energetics

Based on the above data, we hypothesized that the biomechanically triggered shift in cellular energetics would also potentially enable cells to diversify their substrate pool, supporting ATP formation from alternative carbon sources such as lipids and proteins, which may be beneficial in situations of limited nutrient availability such as those typically experienced by cancer cells during metastatic dissemination. Of particular interest was the potential contribution of lipids to cell survival given the role of fatty acid oxidation in the development and progression of cancer has been an area of growing interest in recent years. Indeed, recent reports have suggested that enhanced fatty acid uptake, storage, and oxidation are all key for cancer cell survival, particularly when under high stress conditions such as transiting in the blood during metastatic dissemination.^[^ ^13^, ^27^, ^28^ ^]^

**Figure 5:**
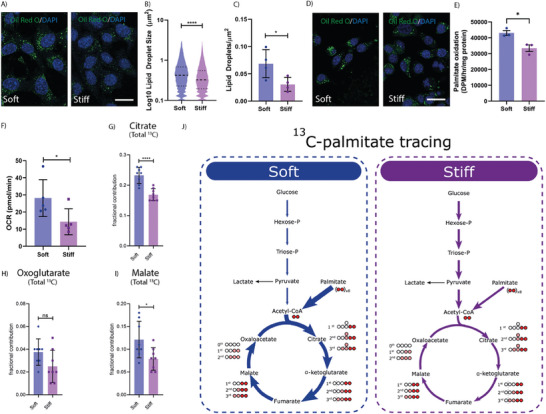
Cells on soft have altered cellular metabolism. A) Representative Oil Red O staining of 4T1 cancer cells cultured directly on soft or stiff microenvironments. Oil Red O (Green) and DAPI (Blue), scale bar = 50 µm. Quantification of B) Lipid droplet size and C) Lipid droplet coverage from the immunostaining images, both indicators of the extent of lipid accumulation within the cytoplasm. Graphs depicts one biological repeat, representative of n = 2 biological repeats. D) Representative Oil Red O staining of E0771 cancer cells cultured directly on soft or stiff microenvironments. Oil Red O (Green) and DAPI (Blue), scale bar = 50 µm. E) Radioactive ^14^C Palmitate experiments demonstrating an increase in palmitate oxidation in the soft primed cells. *n* = 3 biological repeats. Statistical testing using a two‐sided unpaired *t*‐test. F) Quantification of basal respiration values between soft and stiff preconditioned cells, during the fatty acid seahorse stress test. Quantification of the Overall ^13^C enrichment of intermediate abundances of G) Citrate, H) Oxoglutarate, and I) Malate between the soft and stiff conditions. *n* = 4 biological repeats. Statistical testing using two‐sided unpaired *t*‐tests. J) Schematic representation of uniformly labeled C13‐Palmitate tracing study results, depicting an increase in citric acid cycle intermediates in the soft condition. Statistical testing performed using the Mann‐Whitney U test throughout, unless stated otherwise * = *p* < 0.05, **** = *p* < 0.0001.

Oil Red O staining of 4T1 cells biomechanically primed on soft and stiff revealed that soft priming led to an increase in the number and size of intracellular lipid droplets (**Figure** **5**A–C; Figure S5A, Supporting Information). This effect was also observed in E0771 cells which also increased their lipid droplets when primed on soft microenvironments (Figure 5D; Figure S5B, Supporting Information). Assessment of *de novo* fatty acid synthesis enzymes in primed 4T1 cells revealed no significant differences between soft and stiff primed cells (Figure S5C, Supporting Information), suggesting that the increased lipid droplets observed following soft priming are not due to *de novo* synthesis of lipids. In addition, experiments using radioactive ^14^C Glucose and ^14^C Palmitate confirmed that the increased lipid droplets are not due to *de novo* synthesis of lipids, but rather a result of an increase in the accumulation of lipid from extracellular pools. (Figure S5D,E, Supporting Information).

To subsequently assess whether soft primed cells upregulate their fatty acid oxidation machinery, we assessed the capacity for cells to utilize fatty acids to support oxidative phosphorylation in soft and stiff primed cells using a combination of ^14^C Palmitate pulse‐chase studies, and palmitate oxidation studies in the seahorse bioanalyzer. The results from the ^14^C‐Palmitate pulse chase study showed that cells exhibit an increased ability to oxidize exogenous fatty acids when primed on soft microenvironments (Figure 5E). To assess whether this advantage persisted in 3D, and was responsible for the increased colony forming ability of soft cells in in vitro (Figure 2K‐L) and in vivo studies (Figure 3), cancer cells were mechanically primed on either soft or stiff hydrogels and then embedded as single cells into a 3D alginate hydrogel bead. Cell laden 3D alginate beads were then cultured in 0.5 mm glucose media, supplemented only with the fatty acid palmitate. The significantly higher basal respiration values (Figure 5F; Figure S5F, Supporting Information) in the soft primed cells suggests that fatty acid oxidation continues to be significantly more active in this condition, when compared to the stiff primed cells. Further, after the addition of the fatty acid oxidation inhibitor etomoxir, a potent inhibitor of carnitine palmitoyl transferase (CPT‐1), a key enzyme regulating the entry of fatty acids to the tricarboxylic acid (TCA) cycle, only soft primed cells exhibited a response in terms of oxygen consumption rate (OCR), confirming increased utilization of fatty acid oxidation in these cells (Figure S5G,H, Supporting Information).

Finally, ^13^C palmitate mass spectrometry carbon flux tracing analysis of 4T1 cells primed on soft or stiff substrates further confirmed an increase in palmitate oxidation in the soft primed cells. Quantification of ^13^C enrichment into TCA cycle intermediates showed a significant increase in ^13^C Palmitate‐derived m+2 citrate, oxoglutarate and malate, and m+4 citrate and malate in the soft primed cells (Figure S5I–N, Supporting Information), which resulted in a significantly increased overall ^13^C enrichment of both citrate and malate in the soft primed cells (Figure 5G–J). These data together indicate that soft primed breast cancer cells are able to upregulate their fatty acid oxidation capabilities compared to the stiff preconditioned cells.

### 2.6 Biomechanical Priming Alters Intracellular Oxidative Stress

Reports have suggested that the ability of cancer cells to perform fatty acid oxidation is key to their survival, particularly under anchorage independent conditions.^[^ ^75^ ^]^ When cells encounter anchorage independent conditions, such as while transiting in the circulation during metastatic dissemination, all mechanisms of glucose import into the cell are suspended, leading to rapidly depleted ATP stores.^[^ ^76^, ^77^ ^]^ This typically results in increased oxidative stress, often culminating in the activation of apoptotic pathways. This mechanism is thought to be one of the reasons behind the high attrition rate of circulating cancer cells.

**Figure 6:**
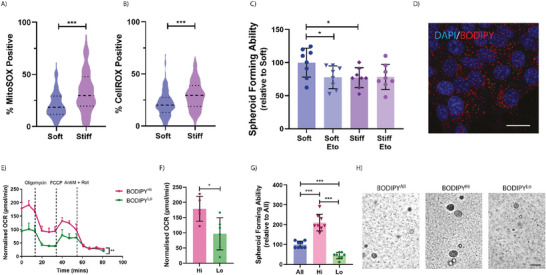
Increased spheroid forming capacity is driven by enhanced fatty acid oxidation. Quantification of the percent A) MitoSOX and B) CellROX positive cells within a population of stiffness preconditioned 4T1 cancer cells embedded in a 3D matrix. Graphs depict one biological repeat, representative of *n* = 3 biological repeats. C) Relative quantification of the spheroid forming ability of stiffness preconditioned 4T1 cells, with and without fatty acid oxidation inhibitor Etomoxir (Eto). Graph depicts one biological repeat, representative of *n* = 3 biological repeats. D) Representative fluorescence image of cells on soft, stained with BODIPY, and counterstained with DAPI. Scale Bar = 25 µm. E) Seahorse bioanalyzer plot of the lipid stress test on BODIPY^Hi^ and BODIPY^Lo^ cell populations. Statistical testing performed using a two‐way ANOVA and F) Quantification of the basal respiration values. G) Relative quantification of the spheroid forming ability of the BODIPY^Hi^, BODPY^Lo^, and BODIPY^All^ populations. Graph depicts one biological repeat, representative of *n* = 3 biological repeats. H) Representative images of spheroid formation at day 5. Scale Bar = 50 µm. Statistical testing performed using the Mann‐Whitney U test throughout, unless stated otherwise * = *p* < 0.05, *** = *p* < 0.001.

The ability for cancer cells to perform fatty acid oxidation is therefore thought to be key to survival. This is in part due to the availability of an alternative fuel source, and second as a mechanism for the regeneration of intracellular antioxidant pools (NADPH), which can help mitigate the effects of cellular oxidative stress.^[^ ^27^, ^61^, ^75^, ^78^ ^]^ To determine whether biomechanical priming altered cancer cell ability to detoxify intracellular reactive oxygen species (ROS), we stained biomechanically primed cells with the intracellular ROS probes mitoSOX and cellROX under conditions of cell detachment. MitoSOX provides a readout of mitochondrial superoxide levels while cellROX provides a broader readout of all cellular reactive oxygen species. Together they can both be used to indicate the levels of oxidative stress within a cell. Our data demonstrate that under experimental conditions of cell detachment, cancer cells that have been primed on soft substrates prior to detachment have significantly lower levels of intracellular ROS. These data indicate that the increase in fatty acid oxidation (FAO) seen in the soft primed cells may be serving to protect cells from oxidative stress (**Figure** **6**A,B).

We next validated whether the enhanced spheroid forming ability of soft primed cancer cells was also dependent on their increased capacity for fatty acid oxidation. By carrying out a spheroid formation assay in the presence of etomoxir (Eto), our data reveal a significantly diminished spheroid forming capacity in soft, but not stiff primed cells in the presence of etomoxir. These data confirm that the spheroid forming advantage imparted by the softer mechanical priming is due, at least in part, to the cells enhanced capacity for fatty acid metabolism (Figure 6C). These data also fit with previously published work which has elegantly demonstrated that etomoxir treatment (to block fatty acid oxidation and reduce metabolic flexibility) of cells that are FAO^high^ shows a reduction in the formation of lung metastasis upon tail vein injection in triple negative breast cancer models.^[^ ^79^, ^80^, ^81^ ^]^

To further determine whether enhanced fatty acid uptake and metabolism are sufficient for spheroid forming ability, we labeled soft primed cells with a live cell lipid droplet tracer (BODIPY™) (Figure 6D), and sorted for the top 10% intensity (BODIPY^Hi^) – representing the cells likely carrying out the highest level of fatty acid oxidation – and bottom 20% intensity (BODIPY^Lo^) – representing the cells likely carrying out the lowest level of fatty acid oxidation – as well as all BODIPY positive cells (BODIPY^All^). When assayed for lipid metabolism in real‐time using the seahorse bioanalyzer, we confirmed that the BODIPY^Hi^ cells were performing significantly higher levels of fatty acid oxidation when compared to the BODIPY^Lo^ cells (Figure 6E,F).

Sorted cells were also embedded as single cells in our 3D spheroid forming assay, where we observed striking differences in the spheroid forming ability. BODIPY^Hi^ cells showed a 4.8‐fold and 2.1‐fold increased spheroids at day 5, compared to the BODIPY^Lo^ and BODIPY^All^ populations, respectively (Figure 6G,H; Figure S6A, Supporting Information). These data confirm that softer microenvironments trigger a shift in the cancer cell metabolic profile toward increased fatty acid metabolism, and that the greater this shift, the greater the spheroid forming advantage conferred to these cells.

Together, these data show that the biomechanical properties of primary tumors play a key role in defining the metabolic profile of cancer cells with important downstream effects on metastatic colonization efficiency and disease progression. While previous studies have established a link between increased tumor biomechanics and increased glycolytic activity,^[^ ^58^, ^82^, ^83^, ^84^ ^]^ both of which frequently associate with poor prognosis in cancer,^[^ ^85^ ^]^ our work joins a concert of recent studies which suggest a more nuanced role of cancer cell metabolism in disease progression, with very context dependent effects at different stages of the metastatic cascade.^[^ ^24^, ^86^, ^87^, ^88^ ^]^

### 2.7 Inhibition of Stiffness Induced Signaling Enhances Cell Fitness

β1 integrin is an essential integrin subunit known to mediate cell adhesion to collagen and represents a critical nexus in cellular mechanotransduction.^[^ ^89^ ^]^ We hypothesized that the observed changes in cellular metabolism may be mediated by specific β1 integrin‐collagen interactions and sought to test this using a β1 integrin inhibitory antibody (β1 iAB). The addition of β1 iAB would block cellular mechanosensing in cells cultured on stiff matrices, causing them to adopt a soft primed phenotype with metabolic changes in lipid metabolism and downstream effects on cell survival and outgrowth.

**Figure 7:**
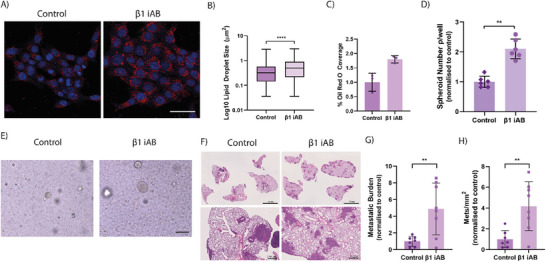
Integrin β1 Inhibitory antibody increases metastatic potential. A) Representative Oil Red O Staining of 4T1 cells on stiff microenvironments, when compared to stiff microenvironments with the inhibitory antibody against Integrin β1 (β1 iAB). Oil Red O (Red) and DAPI (Blue). Scale Bar = 25 µm. Quantification of B) Lipid droplet size and C) ORO coverage in these same conditions. Graphs depict one biological repeat, representative of *n* = 2 biological repeats. D) Quantification of spheroid formation in stiff conditioned cells when compared to stiff conditioned + β1 iAB. Graph depicts one biological repeat, representative of *n* = 3 biological repeats. Statistical testing performed using Mann‐Whitney U test. E) Representative images of spheroid sizes at day 5, comparing spheroid forming efficiency between stiff conditioned control cells and stiff + β1 iAB. Scale Bar = 50 µm. F) Representative images of murine lungs at 2 weeks post injection of stiff control and stiff + β1 iAB cells. Respective quantification of the G) Metastatic burden and H) Number of metastatic lesions per mm^2^ of lung tissue. *n* = 7‐8. Statistical testing performed using two‐sided unpaired *t*‐tests throughout, unless indicated otherwise ** = *p* < 0.01, **** = *p* < 0.0001.

To validate changes in cell behavior, we first assessed the ability for the β1 iAB to alter lipid storage. Our data reveal that β1 iAB treatment of stiff primed cells led to an increase in lipid droplets and droplet size when compared to untreated control (**Figure** **7**A–C), mimicking the observed increase when priming cancer cells on softer microenvironments (Figure 5A).

To determine the downstream, phenotypic effects of β1 iAB treatment, we assessed the 3D in vitro spheroid forming ability of cancer cells primed on a stiff matrix, in the presence or absence of the β1 iAB during the priming stage only. As hypothesised, we observed a significant increase in the spheroid forming ability of the β1 iAB treated cells compared to untreated control, with concomitant increases in spheroid size distribution (Figure 7D‐E; Figure S7A, Supporting Information).

Finally, to determine the in vivo effects of β1 integrin inhibition on metastatic colonization and outgrowth, we performed tail vein injection of stiff primed 4T1 cancer cells, treated with and without β1 iAB during the in vitro priming stage only. Histological staining of harvested lungs showed a striking effect of the β1 iAB, when compared to untreated control (Figure 7F). Quantification of metastases confirmed a significant increase in both metastatic burden and mets mm^−2^ (Figure 7G,H) yet no significant effects on lesion size (Figure S7B, Supporting Information).

These results together show that blocking biomechanical priming, by means of a β1 inhibitory antibody, is sufficient to push cells toward a more metabolically diverse phenotype which in turn provides cells with a greater survival advantage in high stress conditions of metastatic colonization and outgrowth.

## 3 Discussion

Understanding the complex mechanical heterogeneity of the primary tumor microenvironment and the multifaceted role this plays in cancer cell growth, metastatic dissemination, and seeding at secondary sites both in the short‐ and long‐term, is crucial to the development of novel therapeutics for cancer treatment.^[^ ^90^ ^]^ Our work sought to address this by systematically assessing biomechanically primed mammary carcinoma cell behavior and fitness in several in vitro and in vivo models that recapitulate the various stages of the metastatic cascade.

Numerous reports in recent decades have indicated that microenvironmental stiffness, driven by altered matrix remodeling such as increased collagen density, crosslinking, and stiffening^[^ ^1^, ^91^ ^]^ to be critical drivers of disease progression.^[^ ^8^, ^9^ ^]^ Seminal work from almost two decades ago was the first to demonstrate that non tumorigenic cells can acquire tumorigenic properties by simple manipulation of extracellular stiffness.^[^ ^6^ ^]^ Later work from Provenzano et. al. then showed that high density collagen within the mammary fat pad (using Col1a1 mutant mice), associated with increased stiffness, and led to enhanced tumor initiation and progression when compared to the WT mice.^[^ ^92^ ^]^ This malignant transformation in response to stiffness was later attributed to mechano‐activation of the Rho/ROCK pathway,^[^ ^93^, ^94^ ^]^ leading to a multitude of downstream signaling changes including YAP/TAZ translocation and Src driven changes in phosphorylation signaling through a number of cellular pathways.^[^ ^2^, ^95^, ^96^ ^]^ More recently, reports have even uncovered effects of tumor stiffening on stromal cells populations also, with reciprocal pro‐tumorigenic effects upon cancer cells, consequently contributing to disease progression.^[^ ^97^, ^98^ ^]^

Conversely, there have been only a handful of reports that touch on the potentially pro‐tumorigenic effects that soft microenvironments may have on disease progression, citing the enhanced de‐differentiation, tumorigenicity and chemo‐resistance of softer microenvironments on a range of different cancer types in vitro, including breast,^[^ ^59^ ^]^ neuroblastoma,^[^ ^99^ ^]^ hepatocarcinoma,^[^ ^100^ ^]^ melanoma, lymphoma and ovarian cancer.^[^ ^101^, ^102^ ^]^ In this latter work, authors demonstrated enhanced tumorigenicity of the soft cultured melanoma cells in vivo, once injected into the tail veins of mice.^[^ ^101^ ^]^ In the most striking exemplification however, a report from 2014 described the initiation of invasive orthotopic breast tumors, followed by resection and bulk mechanical characterization of the primary tumors and evaluated the knock‐on effects on metastatic burden.^[^ ^30^ ^]^ Following a period of observation, the authors found that the mice that harbored softer, more compliant primary tumors had significantly more and more widespread metastatic lesions, when compared to mice with stiffer primary tumors. The authors hypothesized that the mice with softer tumors were potentially enriched for tumor initiating cells, accounting for a greater local recurrence and metastatic seeding.^[^ ^30^ ^]^ Despite these exciting and significant reports, the specific biological changes that are occurring in response to softer tumor microenvironments, which are in turn driving pro‐tumorigenic cascades in cancer cells, are not well understood. Our study illuminates that biomechanical properties of the primary tumor affect the various stages of the metastatic process differently, and further sheds important light on the pleiotropy of β1 integrin in cancer progression and metastasis.

In this study, we identified significant metabolic changes within mammary carcinoma cells in response to changes in microenvironmental stiffness, with changes in mitochondrial metabolism and substrate utilization. In interrogating the downstream biological effects of these metabolic changes, we found that softer microenvironments are able to equip breast cancer cells with greater survival mechanisms when exposed to high stress conditions in vitro and in vivo. Indeed, our results align with previously published data which have shown that there is a reduction in metastatic seeding when triple‐negative breast cancer cells are treated with inhibitors of mitochondrial metabolism prior to their intravenous injection.^[^ ^87^ ^]^ We further show that soft primed cells not only store significantly more lipids in the form of lipid droplets, but these cells are also more metabolically active, with increased metabolism of lipids via the citric acid cycle. Finally, we link the increased ability for fatty acid oxidation to an enhanced colonization ability. The role of fatty acid oxidation in tumor progression has been increasingly recognized,^[^ ^28^, ^103^, ^104^ ^]^ with many citing the striking pro‐survival mechanisms that are activated in FAO high cells, particularly when under high stress conditions such as anoikis, where cells become increasingly more reliant on non‐glucose sources of ATP and antioxidant production.^[^ ^75^, ^105^, ^106^, ^107^ ^]^

Finally, through using an integrin β1 inhibitory antibody to disrupt cell‐matrix interactions and simulate a softer microenvironment, we demonstrate that we can shift the cellular energetics of cells seeded on stiff matrices to mimic those on softer matrices. These results are in line with others which have found inhibition of β1 integrin to be pro‐metastatic in in vivo models of breast cancer metastasis^[^ ^108^, ^109^ ^]^ although these studies did not look at cellular energetics. However, there have been other reports citing anti‐tumorigenic effects of β1 integrin inhibition in breast cancer.^[^ ^110^, ^111^, ^112^ ^]^ These differences are likely explained by timing of administration and the specific disease stage being studied, with the latter studies focusing primarily on primary tumor growth and proliferation, as opposed to metastatic colonization of secondary sites. Of particular relevance was the observation that while the integrin 1β inhibitory antibody reduced the presence of large, proliferative cell colonies in vitro, the incidence of smaller colonies was three to sixfold higher,^[^ ^111^ ^]^ thus supporting our findings that integrin 1β inhibition leads to enhanced tumorigenicity. Strategies to target β1 integrin therapeutically have not progressed in the clinic, and our work sheds important light on the pleiotropy of β1 integrin in cancer progression and metastasis.

Understanding both the causes and consequences of biomechanical heterogeneity of tumors will allow us to gain a better, more holistic understanding of how tumor ecosystems operate. Our work, and that of others,^[^ ^33^ ^]^ shows that there is a striking biomechanical heterogeneity that exists within a single tumor at any point in time. This biomechanical heterogeneity is sensed by all cells present within the tumor and leads to wide scale cellular reprogramming, that ultimately shapes the evolutionary trajectory of tumor progression. Our work presented herein sheds light on the long‐term effects of biomechanical reprogramming that will influence cancer cell behavior after they leave the primary tumor.

## 4 Experimental Section

### Reagents

All reagents and chemicals were purchased from Sigma‐Aldrich, unless stated otherwise.

### Cell Culture

Triple‐negative murine mammary cancer cells (4T1 Parental, 4T1‐Luc2Tom, 4T1‐Luc, and E0771 Parental) and telomerase immortalized dermal fibroblasts were obtained from ATCC. The E0771‐Luc2GFP cells were made in house, by transfecting E0771 cells with the pFU‐Luc2‐GFP vector, a kind gift from Sam Gambhir, Stanford University. 4T1 cells and their reporter expressing variants, along with the telomerase immortalized dermal fibroblasts were maintained in Dulbecco's modified eagle medium (DMEM) (ThermoFisher Scientific), 10% (v/v) Foetal Bovine Serum (FBS), and 1% (v/v) Penicillin/Streptomycin (P/S) (ThermoFisher Scientific). Cancer cell lines E0771 and E0771Luc2GFP were maintained in Dulbecco's modified eagle medium (DMEM) (ThermoFisher Scientific), 10% (v/v) Foetal Bovine Serum (FBS), 1% L‐Glutamine (ThermoFisher Scientific) and 1% (v/v) Penicillin/Streptomycin (P/S) (ThermoFisher Scientific). Cells were incubated at 37 °C, 5% CO_2_, 21% O_2_. For routine cell culture, cells were washed in Dulbecco's phosphate buffered saline (DPBS) (ThermoFisher Scientific) and detached using 0.25% trypsin‐0.1% EDTA with incubation for 5 min at 37 °C. For detachment from PAGs, a mixture of 0.25% trypsin‐0.1%EDTA/TrypLE (ThermoFisher Scientific) was used.

### Polyacrylamide hydrogels

Polyacrylamide gels were made as previously described^[^ ^113^, ^114^ ^]^ as per the example recipes shown below. Briefly, acrylamide/bis‐acrylamide solutions were prepared in phosphate‐buffered saline without calcium and magnesium (PBS; ThermoFisher Scientific) and de‐gassed under vacuum prior to polymerization. Circular coverslips with a diameter of 50 mm and 13 mm were functionalized using a solution of 3% acetic acid and 0.5% 3‐(trimethoxysilyl)propyl methacrylate, in absolute ethanol for 5 min and allowed to air dry. Large glass slides were treated with dichlorodimethylsilane (DCDMS) to create a hydrophobic surface. The gels were made by adding 0.1% (w/v) freshly made ammonium persulfate (APS) and 0.01% tetramethyl‐ethylenediamine (TEMED) to the polyacrylamide/bis‐acrylamide solution and pipetted between a functionalized coverslip and DMDCS treated glass slide and left to gel for 30 min. Gels were washed in PBS and stored at 4 °C until further use.

**Table:** 

Reagent	Soft gels	Stiff gels
Acrylamide	2.75% Final conc. 343.75 µL	5% Final conc. 625 µL
Bisacrylamide	0.1% Final conc. 250 µL	0.1% Final conc. 250 µL
PBS	4406.25 µL	4125 µL

To enable cell attachment to the surface of the PAGs, we used rat‐tail collagen type 1, made in‐house as previously published.^[^ ^115^ ^]^ Briefly, gels were washed three times with 50 mm HEPES buffer (pH = 8.5), followed by incubation with 0.2 mg mL^−1^ sulfo‐SANPAH (ReAgency) in 50 mm HEPES (pH = 8.5). The sulfo‐SANPAH was then activated with UV light at 365 nm for 10 min using a UV cross‐linker box (Fisher biotec). The hydrogels were then washed twice with 50 mm HEPES (pH = 8.5) and incubated overnight at 4 °C with 0.1 mg mL^−1^ rat‐tail collagen (diluted in 17.4 mm acetic acid). Finally, three 5‐minute washes with Dulbecco's phosphate buffered saline (DPBS) (ThermoFisher Scientific, USA) were completed and the gels were sterilised under UV light for 20 min.

### Unconfined Compression of Hydrogels and Tumor Tissues

Unconfined compression of polyacrylamide hydrogels and tissue chunks was performed on a TA Instruments Dynamic Hybrid Rheometer (DHR‐3, TA Instruments). An 8 mm punch biopsy was taken from the gel/tissue and placed between the upper and lower geometries. A constant compressive rate of 10 mm min^−1^ for hydrogels and 2 mm min^−1^ for tissues was applied to the samples, with data output in the form of axial force (N) and gap (mm). The data was analyzed and a stress/strain curve for each replicate gel was obtained. Stress calculations were corrected for sample size where necessary. Compressive elastic modulus (kPa) was obtained from the linear region of the stress/strain curve. For unconfined compression analysis of the hydrogels, 8 separate PAGs were used. For the measurements of human tissues, 3 tumor chunks, alongside matched healthy tissues (3‐4 per patient) were analyzed.

### Rheology of Hydrogels and Tumor Tissues

Shear rheology of polyacrylamide hydrogels and tissue chunks was performed on a TA Instruments Dynamic Hybrid Rheometer (DHR‐3, TA Instruments). Shear rheology was measured by subjecting samples to a controlled strain with a continuous oscillation and with an oscillation frequency of 0.5 rad per sec; an oscillation strain ranging from 0.2% to 2.0% and an axial force of 0.03 N; a conditioning time of 2.0 seconds and a sampling time of 3.0 s, as previously described.^[^ ^116^ ^]^ Storage modulus was determined as the mean value within the linear viscoelastic range. For rheological analysis of the hydrogels, 9 separate PAGs were used. For the measurements of tissues, 12 tumor chunks, alongside 5 aged‐matched healthy fatpads were analyzed.

### Atomic Force Microscopy of Hydrogels and Tumor Cells

Atomic force microscopy was performed on polyacrylamide hydrogels, using a MFP‐3D atomic force microscope (Asylum Research) as previously described.^[^ ^117^ ^]^ Briefly, 200 µm gold‐coated, silicone‐nitride cantilevers with pyramid‐shaped tips were used at a resonant frequency of 17 kHz and a nominal spring constant of ≈20–25 pN nm^−1^ (PNP‐TR, Nano World). Samples were immersed in 1× PBS without Mg^2+^ and Ca^2+^, indented in triplicate at an approach velocity of 2 µm s^−1^ until a 2 nN trigger force was registered, and then retracted at 10 µm s^−1^. Samples were indented at 500 µm intervals along the gradient axis of polyacrylamide hydrogels. The linear portion of the contact generated force curves was analyzed with custom‐written code in Igor Pro to determine Young's modulus as previously described.^[^ ^118^ ^]^ Six separate hydrogels were measured, with each point representing an average of six separate measurements per hydrogel.

Single‐cell stiffness was measured using the same method as above.^[^ ^119^ ^]^ Indentations were made on the nuclei of the cells over a minimum of four experimental replicates. Nine cells were indented per hydrogel for a minimum of 36 cells examined per experimental condition.

### Brillouin Microscopy of Hydrogels and Tumor Tissues

Confocal Brillouin microscopy was utilized to assess microscopic mechanical properties of polyacrylamide hydrogels, agar and murine tumor tissues as a non‐contact measurement alternative to bulk compression, rheology, and AFM. Briefly, the Brillouin microscopy system comprised of a continuous wave 660 nm laser (Cobolt Flamenco laser), a confocal microscope (CM1, JRS Instruments) and a 6‐pass scanning tandem Fabry–Perot interferometer (TFP1, JRS Instruments). For the measurements of samples, the laser beam was focused into the sample by a long working distance objective lens with 20X magnification (Mitutoyo Plan Apo infinity‐corrected objective, NA = 0.42, WD = 20 mm) and 300 µm aperture was chosen at the spectrometer input to maximize the signal strength and speed up signal acquisition time (20s per point for opaque tumor samples). This resulted in imaging resolution of ≈2 µm × 2 µm × 100 µm in the X‐Y‐Z direction, respectively. After interacting the laser beam with the sample, the light scattered in backwards direction was collected by the same objective lens and redirected to the Brillouin interferometer by the polarization sensitive beam splitter for the detection and analysis. The raw spectra of Brillouin scattered light, containing Rayleigh and Brillouin peaks (Stokes and anti‐Stokes), were fitted using the Damped Harmonic Oscillator model. The exact position of these peaks determines the Brillouin frequency shift (BFS) that is directly proportional to the speed of sound and the longitudinal elastic modulus at GHz frequencies, thus it can serve as a measure of a material's elasticity.^[^ ^38^, ^120^ ^]^ The linewidth (LW) of the Stokes and Anti‐Stokes peaks is determined by the phonon lifetime and generally serves as an indication of: (i) the extent of phonon loss due to viscous energy dissipation and (ii) the level of sample's heterogeneity.^[^ ^38^, ^120^ ^]^ For multicomponent, microscopically heterogeneous tissues such as tumors, the second factor is known to be dominant.^[^ ^121^ ^]^ The exact position of these peaks determines the BFS that is directly proportional to the speed of sound and the longitudinal elastic modulus, thus it can serve as a measure of a material's elasticity.^[^ ^38^, ^120^ ^]^ For the measurements of mechanical properties in polyacrylamide gels we used point measurements owing to homogeneity of the gel material. For the Brillouin mapping of agar and tumor tissues, 2D scan was performed by scanning the samples on the 3D motorized stage along the X‐ and Y‐axis while keeping the optical system and the objective lens stationary. The scanned area was 2 mm × 2 mm, and the scanning step size was set to 100 µm in both directions. Brillouin microscopy data was collected using commercial (Ghost, JRS Instruments) and in‐house built software to perform point measurements and 2D scans of BFS and LW.

### Immunofluorescence

4T1 murine mammary carcinoma cells were seeded onto soft or stiff hydrogels and left to spread for 24 h, after which cells were fixed in 4% paraformaldehyde (Electron Microscopy Sciences) prior to permeabilization with 0.1% Triton‐X in PBS. A blocking buffer of 5% donkey serum and 0.1% Triton‐X in PBS was added to the cells for 2 hours at room temperature. Cell were stained with Phalloidin‐488 (1:250; Thermo‐Fisher Scientific) for 30 minutes, followed by 10 minute DAPI counterstain at 1:1000 (Thermo‐Fisher Scientific) in PBS. Cells were imaged on the Leica DMI 6000 microscope using the 40x objective.

### Cell Cycle Analysis of Cancer Cells Using Click EdU Flow Cytometry Assay

5‐ethynyl‐2 deoxyuridine (EdU) flow cytometry analysis was determined in soft and stiff conditioned 4T1 cells as previously described.^[^ ^122^ ^]^ Briefly, EdU (10 µm) was added to the cells and incubated for 1 h. For negative staining controls, we included DMSO‐treated cells that had not been exposed to EdU. Cells were washed with PBS and trypsinised using TrypLE and inactivated in 0.5 mL of cold FACS Buffer (PBS 1×, 2% FBS v/v, 2 mm EDTA pH 7.9). Cells were fixed in 4% paraformaldehyde and permeabilized in 0.2% (w/v) saponin containing 4% (v/v) FBS (v/v), 1% (w/v) bovine serum albumin (BSA) and 0.02% (v/v) Sodium Azide in PBS prior to EdU detection using an in‐house developed Click‐iT EdU reaction cocktail made of 200 nM AZDye™ 488 Azide (Click Chemistry Tools), 800 µm Copper (II) sulfate, and 5 mm Ascorbic acid in PBS. Following EdU detection, cells were stained with Alexa Fluor 594 anti‐Histone H3 Phospho (Ser10) (Cat: 650 810; Clone 11D8, BioLegend). Alexa Fluor 594 Mouse IgG2b, κ Isotype Ctrl was used as the isotype control (Cat: 400 362; clone MPC‐11, Biolegend). Cells were counterstained with Hoechst 33342. Samples were analyzed by flow cytometry for DNA content and EdU labeled cells using a BD LSR Fortessa Laser Cell Analyser (BD Biosciences, Erembodegem, Belgium). EdU‐AZDye™ 488 Azide and phospho‐Histone H3S10‐Alexa Fluor 594 fluorescence were detected with logarithmic amplification using the B530 (530/30) and YF610 (610/20) detectors, respectively, whereas Hoechst fluorescence was detected with linear amplification using the V450 (V450/50) detector. Data were collected using FacsDIVA 8 software. A minimum of 20000 events were captured per sample with 3 biological repeats performed. Gating strategies are depicted in Figure S8 (Supporting Information). All flow cytometry data were analyzed using FlowJo software (Tree Star Inc.).

### Invasion Assays

Invasion studies into organotypic collagen matrices were performed as previously described. ^[^ ^123^ ^]^ Briefly, 4 × 10^5^ telomerase immortalized dermal fibroblasts were embedded in rat tail collagen I at a final concentration of 2.5 mg mL^−1^. After collagen polymerization, plugs were incubated for 12 days, with media renewal on day 6. Prior to use in invasion assays, pharmacological removal of fibroblasts was achieved with 400 µg/mL hygromycin for 48 h followed by 3 × 30 min washes in PBS, followed by 1 × 30 min equilibration in DMEM with 10% FBS and 1% penicillin/streptomycin to generate a cell‐free matrix that had been remodeled by fibroblasts. Following remodeling, 1 × 10^5^ stiffness preconditioned 4T1 cells were seeded on top of the organotypic matrix and were allowed to settle for 48 h. The organotypic matrix was then transferred to a metal grid establishing an air–liquid interface and cancer cells were allowed to invade for 12 days, with the renewal of DMEM every 72 h. Organotypic matrices were then fixed in 10% formalin and processed for histological analyses. Analysis was performed on three representative regions per organotypic matrix with assessment of invasive depth per cell and number of invaded cells. Cancer cells were considered to have invaded if they were present at a distance of >50 µm from the upper surface. 4 biological repeats were performed.

### Realtime Deformability Cytometry

4T1 cells cultured on soft or stiff PAGs for 24 hours were trypsinized and re‐suspended in CellCarrier (Zellmechanik Dresden) at a concentration of 1–2 × 10^6^ cells mL^−1^. The cell suspension was transferred into a Luer‐Lock syringe and placed in the precision pump of the AcCellerator (Zellmechanik Dresden). After equilibration of the system with CellCarrier buffer and flow stabilization, the deformation of at least 4000 cells in the microfluidic 20 µm channel constriction was analyzed at a flow speed of 0.12 µL s^−1^ and 0.04 µL s^−1^. Cells obtain a bullet‐like shape in the channel constriction and are characterized by their deformation (D), defined as the deviation from a perfect circle (D = 1‐ c). As reference, circularity of the cell lines was also recorded in a section of the microfluidic chip with wider cross section (reservoir). During the deformation cytometry, data was recorded in ShapeIn and subsequently analyzed using ShapeOut (Zellmechanik Dresden). Events outside the size range of 85–800 µm^2^ or with an aspect ratio exceeding 2.0 were excluded from the datasets. 3 biological repeats were performed.

### Fluid Shear Stress Assays

4T1 cells were cultured on soft and stiff PAGs for 24 h, prior to trypsinization and subject to shear stress, as previously described.^[^ ^114^ ^]^ Briefly, cells resuspended at a concentration of 5 × 10^5^ cells mL^−1^ in DMEM and exposed to five manual repeated passages of shear stress through a 30‐gauge needle at a constant flow rate of 100 µL s^−1^. Here, Poiseuille's equation was used to measure shear stress, τmax = 4Qη/πR3, whereby *Q* is the flow rate (0.1 cm^3^ s^−1^), *η* is the dynamic fluid viscosity of the cell culture medium at room temperature (0.78 × 10^−3^ N s m^−2^), and *R* is the radius of the needle (R = 7.94 × 10^−3^ cm), resulting in τmax = 2500 dyne cm^−2^.^[^ ^124^ ^]^ The unsheared control cells were not subject to any manipulation. After exposure to shear stress, 5 × 10^5^ cells were seeded back onto stiff PAGs for 24 h. Following trypsinization, cell death at 24 h post shear stress was assessed by flow cytometry using annexin V (fluorescein isothiocyanate)/PI staining kit on unfixed cells as per the manufacturer's instructions. Flow cytometric detection of the annexin and PI was performed using the FACSCanto II (Becton Dickinson Biosciences) with gating strategies depicted in Figure S9 (Supporting Information). Quantification was performed in FlowJo software (Tree Star Inc.). A minimum of 50 000 events were analyzed per sample and 3 biological repeats.

### Spheroid formation in Alginate Hydrogels

Alginate hydrogels were prepared as previously described.^[^ ^125^ ^]^ Briefly, a stock solution of 2% (w/v) alginate (Novamatrix, Norway) in 0.9% saline was prepared and filter sterilized using a 0.4 µm syringe filter unit. For cell embedding, a final alginate suspension containing 1% (w/v) alginate solution, 1 mg mL^−1^ rat tail collagen, 4.6 µm NaOH, 5 mm CaCO_3_ and cells at a concentration of 1000 cells per plug was prepared. Finally, D‐(+)‐Gluconic acid δ‐lactone (GDL) at 0.42% dissolved in 0.9% saline was added to the alginate/cell mixture. The gels were allowed to set for 30 minutes until they became transparent. Gels were then washed twice with PBS and replaced with normal culture media. For all studies, cells were preconditioned on soft or stiff PAGs for 24 h, prior to their trypsinization and embedding into the alginate hydrogels. For the integrin 1β inhibition studies, cells were cultured for 24 h on stiff hydrogels, with 20 µg mL^−1^ of integrin 1β inhibitory antibody (Clone 9EG7; BD Bioscience Cat: 553 715). Control condition for this study was cells on stiff, without antibody. In the respective experiments, cells were treated with 0.5 nm rotenone or 75 µm etomoxir, immediately after alginate hydrogel gelation and for the duration of the experiment. Spheroid size and number measurements were performed on days 5, 8, and 12 by imaging three regions of interest per hydrogel, with a minimum of six hydrogels per condition. 3–4 biological replicate experiments were performed for each of the spheroid formation experiments.

### PI/Hoesct Staining

Cell laden alginate hydrogels were prepared as described above, at a density of 1.5 × 10^5^ cells per 100 µL. For PI/Hoechst staining, hydrogels were washed twice with PBS prior to incubation with a solution of 2 µg mL^−1^ Propidium Iodide (ThermoFisher Scientific) in media for 30 min. Hydrogels were washed with PBS and prepared for imaging in media, supplemented with 250 ng mL^−1^ Hoechst33342 (Thermo‐Fisher Scientific) for detection of all cell nuclei. Imaging was performed on the Thermo ArrayScan VTI high content microscopic imager, imaging 20 regions of interest per hydrogel, and a minimum of six replicate gels per condition. *n* = 3 biological repeats.

### Barcoding of 4T1 Cancer Cells

Barcoded 4T1 cells were a gift from Dr Simon Junankar.^[^ ^41^ ^]^ A barcoded population of 4T1 murine mammary carcinoma cells, containing 5000 individual barcodes, were cultured on soft and stiff hydrogels for 6 days, with cell passaging on days 1 and 3. Cells were harvested for DNA extraction (Qiagen) on days 1, 3, and 6. All samples underwent targeted barcode polymerase chain reaction (PCR) amplification according to the updated version of the original protocol^[^ ^40^ ^]^ available on the Addgene website (https://www.addgene.org/pooled‐library/clontracer/). Specific PCR products (180 bp) were gel purified, quantified by Qubit 2.0 fluorometer (ThermoFisher Scientific) and pooled into a library. Prior to sequencing, an equal combination of additional PCR products containing two inverse barcodes (GACTCAGTGTCAGACTGAGTGTCTGACTGT and CTGAGTCACAGTCTGACTCACAGACTGACA) plus the PhiX Control V3 (Illumina, CA, USA) were spiked in to balance the nucleotide distribution within the library. Samples were sequenced using a custom sequencing primer (GCGACCACCGAGATCTACACACTGACTGCAGTCTGAGTCTGACAG) and the NextSeq 500/550 Mid Output Kit v2 – 150 cycles (FC‐404‐2002, Illumina, CA, USA) on the Illumina NextSeq platform. Barcode composition analysis and calculation of barcode overlap between samples were performed as indicated in the original protocol^[^ ^40^ ^]^ and updated Python scripts available from the Addgene website (https://www.addgene.org/pooled‐library/clontracer/). Data is depicted as the barcode diversity in each condition, when compared to the tissue culture plastic (TCP) control sample. *n* = 3 biological repeats.

### In Vivo Studies

Study approval was obtained from the St Vincent's Clinic Precinct Animal Welfare Committee (protocol numbers 17/23, 19/08, and 22/04). Experiments were conducted in accordance with the Australian Code of Practice for the Care and Use of Animals for Scientific Purpose. For the single stiffness preconditioning studies, 4T1‐Luc2Tom, 4T1 parental, or E0771–Luc2GFP murine mammary carcinoma cells were cultured of soft or stiff hydrogels for 24 hours. For the flip stiffness study, 4T1‐Luc cells were cultured on the soft or stiff hydrogels for 24 h, followed by trypsinization and subsequent seeding on soft or stiff hydrogels for a further 24 h period. For the integrin 1β inhibition studies, cells were cultured for 24 h on stiff hydrogels, with 20 µg/mL of integrin 1β inhibitory antibody (Clone 9EG7; BD Bioscience Cat: 553 715). Control condition was cells on stiff, without antibody. Cells were detached from the hydrogels by using a mixture of 0.25% trypsin‐0.1% EDTA/TrypLE (ThermoFisher Scientific). For animal injections, cells were resuspended in HBSS (Gibco) at a concentration of 1 × 10^5^ cells (2 × 10^5^ cells for Int1β study) in 100 µL and kept on ice. 100 µL of the single cell suspensions were tail vein injected into BALB/cJAusb (4T1 model) or C57BL/6 (E0771 model) mice (*n* = 6–10 per group). At each experimental time point (2, 24, and 48 h for time course study; 2 weeks for the Int1β study and 3 weeks for all other studies), mice were sacrificed and their lungs perfused with 3% formaldehyde, 60% ethanol, 4% acetic acid in water to ensure adequate inflation and in situ fixation. Lungs were then processed for histological analysis. For the generation of orthotopic tumors for biomechanical characterization, 1 × 10^5^ 4T1 cells were injected into the fat pad of Balb/C mice with tissue harvesting one tumors reached appropriate size.

### Human Tissue Collection

This study was carried out in strict accordance with the approved protocol and good clinical practice standards. The study protocol was reviewed and approved by the St Vincent's Human Research Ethics Committee (Project SHARE, 2019/ETH03101). All patients provided written informed consent before undergoing study‐specific procedures.

### Histological Analysis

Fixed lung tissues were prepared for histological analysis by paraffin processing and embedding (Leica Peloris II). In order to ensure adequate sampling of the lung tissues, three levels (250 µm apart) were taken from each lung for subsequent histological analysis. Sections were deparaffinised and stained following standard Hematoxylin and Eosin procedures on the Leica ST5010 Autostainer XL (Australian Biostain, Harris non‐toxic (acidified) and Eosin Phloxine Alcoholic 1%). Stained slides were imaged at 20x magnification (Aperio Scanscope). Image processing was performed on QuPath where each metastatic foci was detected and the area measured. Results shown represent metastatic burden (area of all metastatic lesions/area of lung tissue), mets mm^−2^ (number of metastatic lesions/area of lung tissue), and metastasis size (size of each metastatic lesion).

### Multiplex qPCR for Vimentin and Luciferase

At the time of harvest, mice were euthanized and a single lobe of the mice lungs was excised and snap frozen prior to perfusion and fixation. Frozen samples were digested in a solution of 100 mm NaCl, 10 mm Tris HCl, 25 mm EDTA, 5% SDS, supplemented with proteinase K (Thermo‐Fisher Scientific), overnight at 55 °C. The digested lung tissues were subject to protein precipitation using a high concentration salt solution. Supernatant was then subject to a phenol/chloroform/IAA (Thermo‐Fisher Scientific) DNA extraction, obtaining whole cell genomic DNA (gDNA). A quantitative multiplex PCR was performed on the gDNA using Luciferase (Cat: 4 331 182; Thermo‐Fisher Scientific) and mouse Vimentin (Cat:4 448 489; Thermo‐Fisher Scientific) TaqMan probes and primers, as per manufacturers recommendations on QuantStudio 7. Mouse vimentin expression was used for normalization of reactions prior to calculations to quantitate gene expression using the comparative CT method.^[^ ^126^ ^]^ Metastatic burden was expressed as a fold‐change as calculated by 2‐X with X = (Animal 1 organ mLuciferase CT – Animal 1 organ mVim) – (Animal 2 organ mLuciferase CT – Animal 2 organ mVim), with Animal 1 being the animal to which all samples are normalized.

### Glucose Biosensor FRET Imaging

The glucose FRET biosensor with mTurquoise2 and sReACh combination was a gift from Professor Erik Sahai.^[^ ^66^ ^]^ FRET biosensor stably‐expressing 4T1 mammary carcinoma cell line (4T1 sReACh) was made using the PiggyBac transposon system. DNA plasmids were transfected using Lipofectamine 2000 reagent (Thermo‐Fisher Scientific). FRET biosensor‐expressing cells were sorted by BD fluorescence‐activated cell sorting (FACS) Aria III for the mTurquoise2 fluorescence and a pure population was generated. 4T1 sReACh cells were seeded on soft and stiff hydrogels. Imaging of the mTurqouise2‐sReACh Glucose‐FRET biosensor^[^ ^66^, ^127^ ^]^ was performed as described previously^[^ ^128^, ^129^, ^130^ ^]^ on a Leica DMI 6000 SP8 confocal microscope using a 25× 0.95 NA water immersion objective on an inverted stage. The Ti:Sapphire femtosecond laser (Coherent Chameleon Ultra II, Coherent) excitation source operating at 80 MHz was tuned to a pumping wavelength of 840 nm. A RLD‐HyD detector was used with a 483/40 nm bandpass emission filter to detect mTurquoise2. Images were acquired at a line rate of 700 Hz, 512 × 512 pixel, and at a total of 203 frames per image. Realignment of the data was performed using Galene (v2.0.2^[^ ^131^ ^]^) using the warp realignment mode, 10 realignment points, a smoothing radius of 2px, and a realignment threshold 0.6 for the mTurqouise2 signal. Single cell analysis was performed using FLIMfit (v5.1.1^[^ ^132^ ^]^) by drawing ROIs encompassing the cytoplasm. A reference signal to estimate the IRF was acquired using Atto 425 dye diluted 1:1000 in H_2_O. *n* = 3 biological repeats.

### Mitochondrial Staining

4T1 murine mammary carcinoma cells were seeded on soft and stiff polyacrylamide hydrogels. After 24 hours, cells were gently washed with PBS, followed by incubation with MitoTracker Orange (Thermo‐Fisher Scientific) or JC‐1 (Thermo‐Fisher Scientific) for 30 min at 37 °C. Cells were washed with PBS prior to imaging on the Leica DMI 6000 microscope (40x objective). Image processing was performed on ImageJ in a minimum of three ROIs per replicate, with three replicate gels per experiment (*n* = 2 biological repeats). Fluorescence for both stains was quantified as integrated density per cell. For flow cytometric analysis of these mitochondrial specific dyes, cells were trypsinized from the polyacrylamide hydrogels and stained as a single cell suspension. Cells were resuspended in PBS + 3% FBS for flow cytometric detection of YG582/15 (Mitotracker Orange) and V610/20A (JC‐1) on the BD fluorescence‐activated cell sorting (FACS) Aria III with gating strategies depicted in Figures S10 and S11 (Supporting Information). Mitotracker orange staining was performed on cells from three independent hydrogels, with two biological repeats. Analysis of the flow cytometric data assessed proportion of cell population that was above a pre‐determined mitotracker orange threshold. For analysis of the JC‐1 data, cells were binned into quantiles with respect to their signal in the V610/20A channel. JC‐1 staining was performed on cells from four independent hydrogels, with two biological repeats.

### Densitometry Studies for Fatty Acid Synthesis Enzymes

4T1 cells were cultured on soft or stiff PAGs for 24 h, after which cells were washed with ice‐cold PBS and lysed using a standard lysis buffer (50 mm Tris HCl pH 7.4, 150 mm NaCl, 1 mm EDTA, 1% (v/v) Triton X‐100) containing protease inhibitor (Roche) and 0.2 mm sodium orthovanadate. Lysate samples were quantified for protein content and 10 µg protein was separated on a 10% SDS‐PAGE gel before being transferred onto 0.2 µm PVDF membranes for immunoblotting. Primary antibodies used included phosphorylated (Ser79) acetyl‐CoA carboxylase (ACC, Cat: #3661 Cell Signaling Technology), ACC (Cat: #3662, Cell Signaling Technology), FAS (Cat: #3180 Cell Signaling Technology) with β‐Actin (Cat: sc47778, Santa Cruz Biotechnology) as a loading control. Densitometry was performed and band intensities were calculated using Image Lab (Bio‐rad) and are represented as normalized to the mean of respective controls. *n* = 3 biological repeats.

### Seahorse Bioanalyzer Stress Tests

For measurement of oxidative metabolism in real time, soft and stiff preconditioned cells were trypsinized and resuspended in 1% alginate (Novamatrix, Norway) in 0.9% saline at a density of 4 × 10^4^ cells per 10 µL bead. Cell laden beads were gelled by submersion in a bath of 75 mm CaCl_2_ for 10 min, after which beads were transferred into complete media. Only the BODIPY^Hi/Lo^ seahorse assay was performed on adherent cells (5 × 10^4^ cells per well). At the start of the assay, a single bead was added per well and medium was replaced with 500 µL of fresh seahorse assay media containing 25 mm glucose, 4 mm glutamine and 1 mm pyruvate in DMEM. Mitochondrial metabolism was assayed in a Seahorse XF‐24 extracellular flux analyzer, as per manufacturers recommendations, including addition via ports A–D of 2 µm oligomycin, 1 µmFCCP, and 0.5 µm rotenone with 0.5 µm antimycin A. For assaying fatty acid oxidation capacity, cell laden alginate beads were exposed to a substrate limited media for 6 h, containing 0.5 mm glucose, 1 mm glutamine 1% FBS and 0.5 mm L‐carnitine in DMEM. Immediately prior to the assay, media was replaced with the assay media containing 2 mm glucose, 0.5 mm L‐carnitine and 200 µm of BSA conjugated palmitate (Agilent) in DMEM. The additions on ports A–D were performed using 4 µm etomoxir, 2 µm oligomycin, 1 µm FCCP, and 0.5 µm rotenone with 0.5 µm antimycin A. All inhibitors were prepared in ethanol. Each measurement cycle is represented by a time point in the raw trace data in the figures. For calculation of basal respiration, maximal respiration, proton leak, ATP synthesis, and spare capacity, data points represent individual wells, where average values were calculated from the raw trace data. For the BODIPY^Hi/Lo^ experiment, where cells were adherent, OCR data was normalized to protein content, as measured via BCA, as per manufacturer's recommendations (Pierce™).

### Radioactive ^14^C Glucose and ^14^C Palmitate Experiments

To assess de novo lipogenesis, cells were incubated for 2h in low glucose DMEM containing ^14^C‐glucose (2µCi mL^−1^) while cultured on soft or stiff hydrogels. At the conclusion of the assay the media was acidified with 1 mol L^−1^ perchloric acid and lipogenesis rate was calculated from the incorporation of ^14^C glucose carbons into the cellular lipid pool following a Folch extraction.^[^ ^133^ ^]^ Measurement of the lipid uptake and subsequent utilization was undertaken through a pulse‐chase experiment. Briefly, cells were incubated in low glucose DMEM containing 200 µmol L^−1^ palmitate (conjugated to 1% BSA) and 1‐[^14^C]‐palmitate (1.5µCi mL^−1^) for a period of 4 h. After the priming period, cells were washed and lipid uptake and esterification determined as the amount of 1‐[^14^C]‐palmitate incorporated into the lipid pool following a Folch extraction. A second group of cells that had been pre‐labeled with 1‐[^14^C]‐palmitate for 4h was washed, fresh media (low glucose DMEM) applied for 2h and the oxidation of labeled cellular lipids was assessed by measuring the appearance of [^14^C]‐carbon both in released CO_2_ and in the aqueous fraction of cells that had undergone lipid extraction (incomplete oxidation metabolites). *n* = 3 biological repeats.

### Oil Red O Staining

4T1 and E0771 murine mammary carcinoma cells were seeded on soft and stiff polyacrylamide hydrogels. For the integrin 1β inhibition studies, cells were cultured for 24 hours on stiff hydrogels, with 20 µg mL^−1^ of integrin 1β inhibitory antibody (Clone 9EG7; BD Bioscience Cat: 553 715). Control condition for this study was cells on stiff, without antibody. After 24 h, cells were washed with PBS prior to fixation in 4% paraformaldehyde (Electron Microscope Sciences) for 30 min. Cells were washed 2x with MilliQ water prior to a 5 min incubation in 60% isopropanol. Cells were stained in a 0.3% solution of Oil Red O in Isopropanol for 15 min. Cells are washed 5x with MilliQ water and counterstained with DAPI. Cells were imaged immediately on the Leica DMI 6000 microscope, with an Excitation wavelength of 647, using the 40x objective. Data analysis was performed on three images per hydrogel, with three hydrogels per condition using ImageJ. Analysis of the oil droplet size and number per cell area was performed. *n* = 2 biological repeats for both cell lines.

### BODIPY Staining and Sorting of Cells

4T1 murine mammary carcinoma cells were seeded on soft polyacrylamide hydrogels. After 24 h, cells were stained for 30 min with a 1 µm solution of BODIPY (ThermoFisher), as per manufacturer's recommendations. After staining, cells were trypsinized and prepared for cell sorting in a buffer of PBS, 3% FBS, 1% pen/strep and 0.5 mm EDTA. Cells were sorted based on BODIPY intensity in the B‐530A channel (BD Aria III Cell Sorter). Dead cells were excluded based on DAPI intensity in the V‐450A channel. BODIPY^Hi^ populations were derived from the cells in the top 10% staining intensity, while the BODIPY^Lo^ cells, from the bottom 20%. BODIPY^All^ cells were collected from all the live cells within the population. Gating strategy depicted in Figure S12 (Supporting Information). Cells were sorted into a buffer of PBS with 20% FBS and 3% pen/strep and used for spheroid formation in alginate hydrogels, or for seahorse bioanalyzer assays.

### MitoSOX/CellROX Assays

4T1 murine mammary carcinoma cells were cultured on soft and stiff hydrogels for 24 h. Cells were trypsinized with a solution of 0.25% trypsin‐0.1%EDTA/TrypLE (ThermoFisher Scientific) and embedded into alginate at a density of 20 000 cells per 10 µL and left to equilibrate in complete media overnight. Staining with the MitoSOX and CellROX probes (Thermo‐Fisher Scientific) was performed as per manufacturer's recommendations. Briefly, for staining with MitoSOX, cell plugs were washed with PBS, prior to incubation in a 5 µm MitoSOX probe in HBSS for 10 min. Cells were washed with PBS, counterstained with 250 ng mL^−1^ Hoechst33342 and imaged on the Thermo ArrayScan VTI high content microscopic imager, imaging 20 regions of interest per hydrogel, and a minimum of six replicate gels per condition. For staining with CellROX, staining was performed at 5 µm CellROX in media, and incubated for 30 minutes. Cell plugs were washed with PBS, counterstained with 250 ng mL^−1^ Hoechst33342 and images taken, as described above. Positive events were thresholded within each replicate assay and applied to all conditions/technical replicates in each replicate experiment. Staining was performed using 6–12 technical repeats per biological repeat. Data depicts percentage of MitoSOX or CellROX positive cells within the total population. *n* = 2 biological repeats.

### ^13^C Palmitate Tracing Studies

^13^C Palmitate tracing studies were performed as so to mimic the experimental conditions of the palmitate stress test. As such, cells were conditioned on soft or stiff hydrogels for 24 h, followed by a 6 h incubation in substrate limiting media (DMEM with glucose [0.5 mm], glutamine [1 mm], dialyzed serum [1%], L‐Carnitine [0.5 mm] and pen/strep [1%]) and a final 4 h pulse in assay media containing the uniformly labeled C13‐Palmitate (Cambridge Isotype Laboratories), conjugated to 2% lipid free BSA (DMEM with glucose [2 mm], L‐Carnitine [0.5 mm], pen/strep [1%] and uniformly labeled C13 Palmitate [200 µm]). For mass spectrometric analysis, cells were washed with PBS, followed by gentle cell scraping in PBS. Intracellular metabolites were then extracted using an extraction buffer of acetonitrile:MeOH:H_2_O (4:4:2 ratio), spiked with an internal standard (Thymind‐d4). Supernatants were then collected and dried by speedvac (ThermoScientific Savant SpeedVac SPD140DDA) for 4 hours without heat. Dried lysates were then resuspended in equal volumes of buffer A (20 mm ammonium hydroxide, 20 mm ammonium acetate, 5% acetonitrile), and buffer B. Samples were analysed by LC‐MS, run on a Shimadzu‐QTRAP6500+. LC separation was achieved using an Agilent Poroshell 120 HILIC‐Z column (2.1 × 100 mm, 2.7 µm). The injection volume was 5 µL. Data extraction and analysis were performed using MSConvert and MATLAB. Intensities normalized to the internal standard. *n* = 4 biological repeats.

### Statistical Analysis

GraphPad Prism v9 was used for all statistical analyses. Summary data in figures are presented as mean with standard deviation unless otherwise stated. Sample size and statistical methods varied throughout and are described for each experiment separately within the respective figure legends. Asterisks denote statistical significance, * = *p* < 0.05, ** = *p* < 0.01, *** = *p* < 0.001, and **** = *p* < 0.0001.

## Conflict of Interest

The authors declare no conflict of interest.

## Supporting information

Supporting Information

## Data Availability

The data that support the findings of this study are available from the corresponding author upon reasonable request.
