# Supplementary material for: Tumor Biomechanics Alters Metastatic Dissemination of Triple Negative Breast Cancer via Rewiring Fatty Acid Metabolism
Source: Adv Sci (Weinh). 2024 Apr 11;11(23):2307963. doi: 10.1002/advs.202307963 (PMC11186052; doi:10.1002/advs.202307963)
Supplement: Supplementary file 1 — Supporting Information [file ADVS-11-2307963-s001.pdf]

ADVANCED SCIENCE
Open Access

Supporting Information

for *Adv. Sci.*, DOI 10.1002/advs.202307963

Tumor Biomechanics Alters Metastatic Dissemination of Triple Negative Breast Cancer via Rewiring Fatty Acid Metabolism

Elysse C. Filipe, Sipiththa Velayuthar, Ashleigh Philp, Max Nobis, Sharissa L. Latham, Amelia L. Parker, Kendelle J. Murphy, Kaitlin Wyllie, Gretel S. Major, Osvaldo Contreras, Ellie T. Y. Mok, Ronaldo F. Enriquez, Suzanne McGowan, Kristen Feher, Lake-Ee Quek, Sarah E. Hancock, Michelle Yam, Emmi Tran, Yordanos F. I. Setargew, Joanna N. Skhinas, Jessica L. Chitty, Monica Phimmachanh, Jeremy Z. R. Han, Antonia L. Cadell, Michael Papanicolaou, Hadi Mahmodi, Beata Kiedik, Simon Junankar, Samuel E. Ross, Natasha Lam, Rhiannon Coulson, Jessica Yang, Anaiis Zaratzian, Andrew M. Da Silva, Michael Tayao, Ian L. Chin, Aurélie Cazet, Maya Kansara, Davendra Segara, Andrew Parker, Andrew J. Hoy, Richard P. Harvey, Ozren Bogdanovic, Paul Timpson, David R. Croucher, Elgene Lim, Alexander Swarbrick, Jeff Holst, Nigel Turner, Yu Suk Choi, Irina V. Kabakova, Andrew Philp and Thomas R. Cox**

Supporting Information:

Filipe *et al.*

Tumor Biomechanics Alters Metastatic Dissemination of Triple Negative Breast Cancer via Rewiring Fatty Acid Metabolism

Figure S1

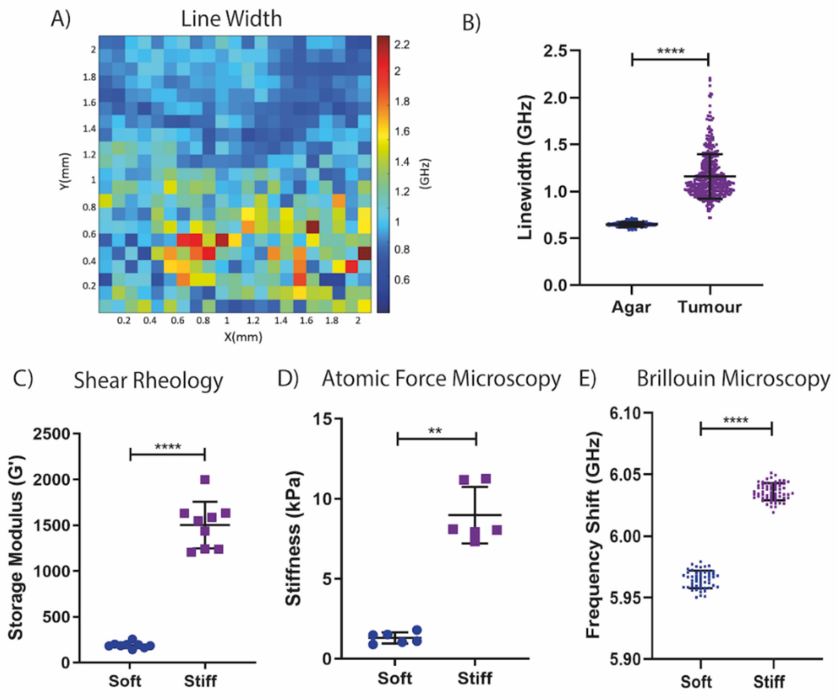

***A**) 2D heat map of the Brillouin line width across the surface of a tumor sample, with LW variation indicating heterogeneity and distribution of viscoelasticity across the sample. **B**) Representation of the line width values across a single tumor, when compared to the control material, agar. Biomechanical profiling of the soft and stiff polyacrylamide hydrogels using a range of different approaches. **C**) Shear Rheology, providing data of the storage modulus (G'), n=9, **D**) Atomic Force Microscopy, yielding stiffness information (kPa; n=6) and **E**) Brillouin frequency shift as detected by Brillouin Microscopy measures the interaction of photons with high frequency pressure waves in the hydrogel material (GHz; n=2. Statistical testing performed using the Mann-Whitney U test throughout, ** = p<0.01, **** = p<0.0001.*

Figure S2

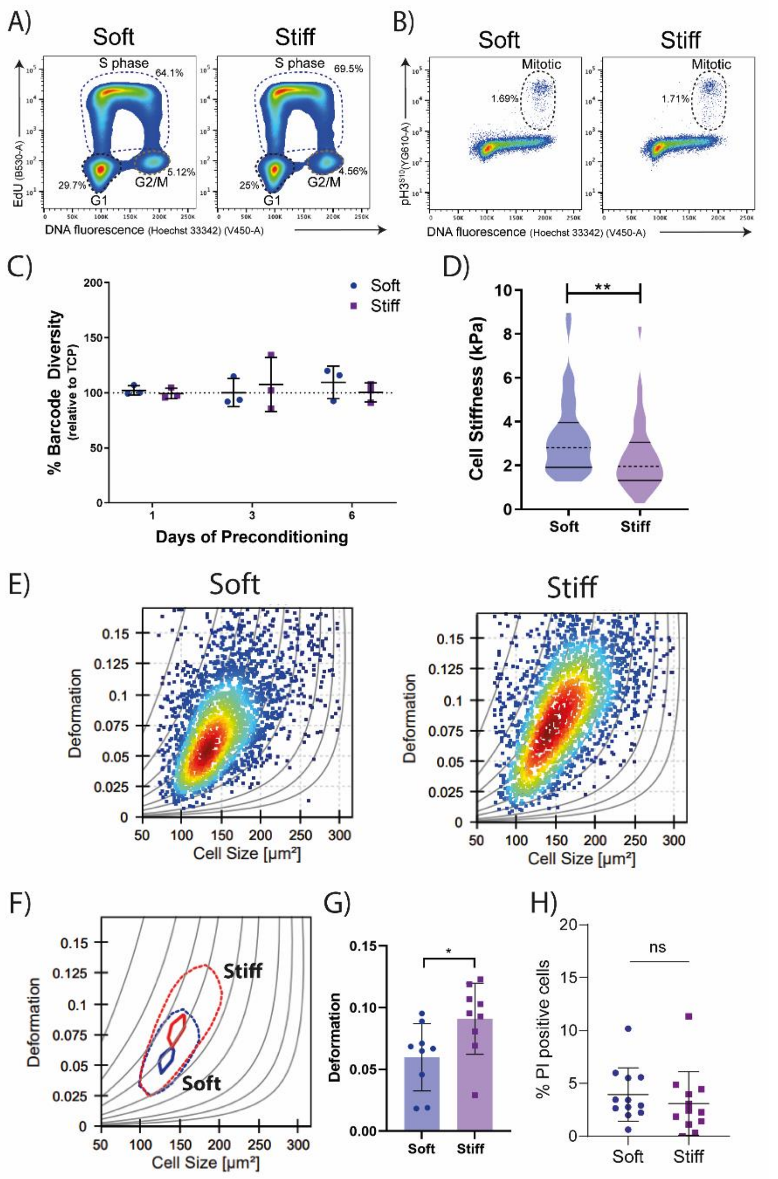

***A**) Representative flow cytometry plot of the EdU incorporation after a single 1hr pulse with EdU monomer and **B**) proportion of pHistone3 positive cells whilst on soft or stiff conditions. **C**) Relative quantification of the barcode diversity within a population of genetically barcoded 4T1 mammary carcinoma cells cultured on soft and stiff microenvironments for 6 days. n=3 biological repeats. **D**) Single cell AFM measurements on cells primed on soft or stiff PAGs for 24 hours. Data obtained from n=5 independent hydrogels. **E**) Representative plot of cell deformability of soft and stiff primed cells. **F**) Population plot of cell deformability on these conditions. **G**) Quantification of n=3 biological repeats performed. **H**) Quantification of Propidium Iodide (PI) positive cells in a population of stiffness preconditioned 4T1 cells, embedded as single cells within a three dimensional matrix. Graph depicts one biological repeat, representative of n=3 biological repeats. Statistical testing performed using the Mann-Whitney U test throughout, * = $p<0.05$, ** = $p<0.01$.*

Figure S3

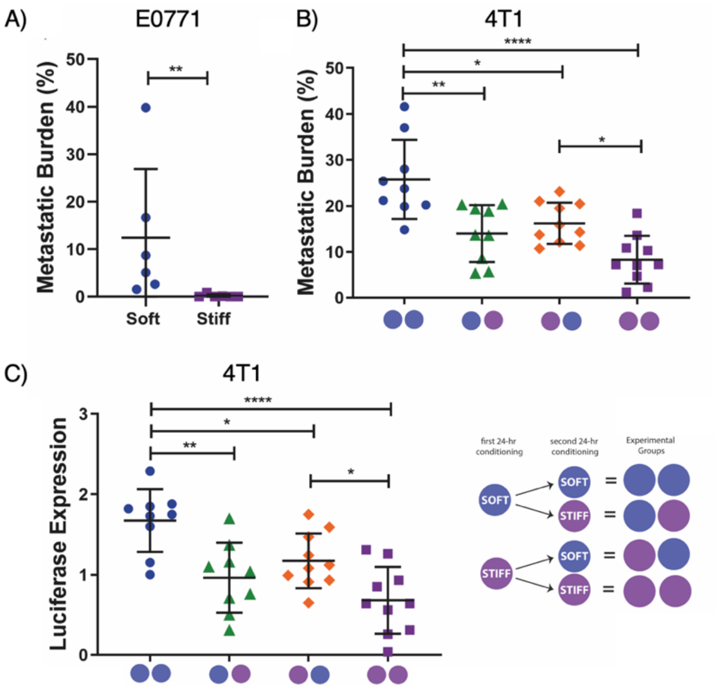

*__A__) Quantification of metastatic burden at 3 weeks post intravenous injection of stiffness preconditioned E0771 mammary carcinoma cells. Quantification from 3 stepped sections per mouse, n=6 mice per group. Statistical testing performed using the Mann-Whitney U test. __B__) Quantification of metastatic burden at 3 weeks post intravenous injection of 4T1 mammary carcinoma cells, preconditioned as per panel C [right]. Quantification from 3 stepped sections per mouse, n=9-10 mice per group. Statistical testing performed using a one-way ANOVA. Quantification performed from the histological sections of lung tissue and __C__) Quantification performed from multiplex qPCR of cancer cell burden/all cells. Statistical testing performed using a one-way ANOVA. * = p<0.05, ** = p<0.01, **** = p<0.0001.*

Figure S4

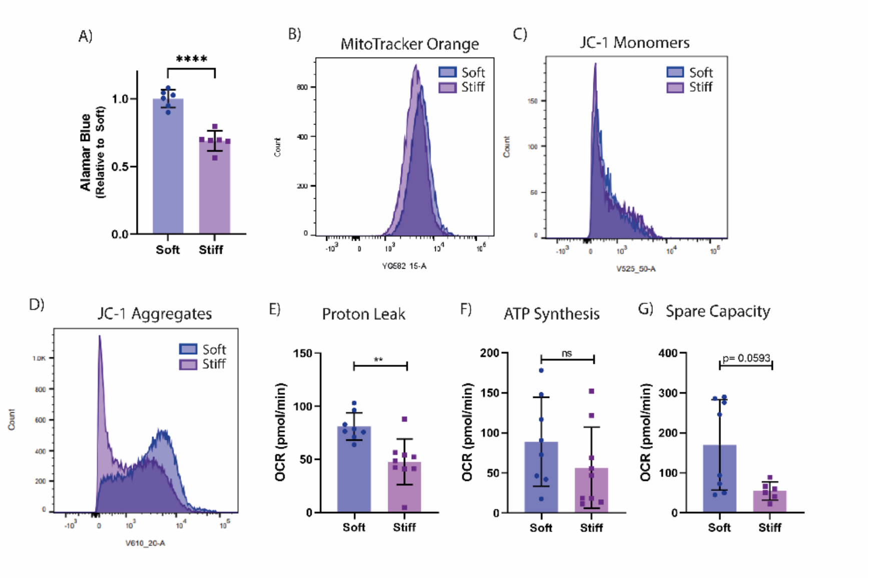

***A**) Day 0 Alamar Blue readings from cells primed on soft or stiff PAGs, embedded as single cells into alginate hydrogels. Graph depicts one biological repeat, representative of n=2 biological repeats. Statistical testing performed using a two-sided unpaired t-test .**B**) Representative flow cytometry histogram of stiffness preconditioned 4T1 mammary carcinoma cells, stained with MitoTracker orange dye. Representative flow cytometry histogram of **C**) JC-1 monomers and **D**) JC-1 aggregates of stiffness preconditioned 4T1 cells, stained with JC-1 dye. Quantification of **E**) Proton leak, a measure of remaining basal respiration, not coupled to ATP production **F**) ATP synthesis, which shows the ATP produced by the mitochondria that contributes to meeting the energetic needs of the cells and **G**) Spare capacity, a measure of a cells capacity to respond to energetic demand, in stiffness preconditioned 4T1 cells when measured by seahorse bioanalyzer. Statistical testing performed using the Mann-Whitney U test throughout, unless stated otherwise. ** = p<0.01, **** = p<0.0001.*

Figure S5

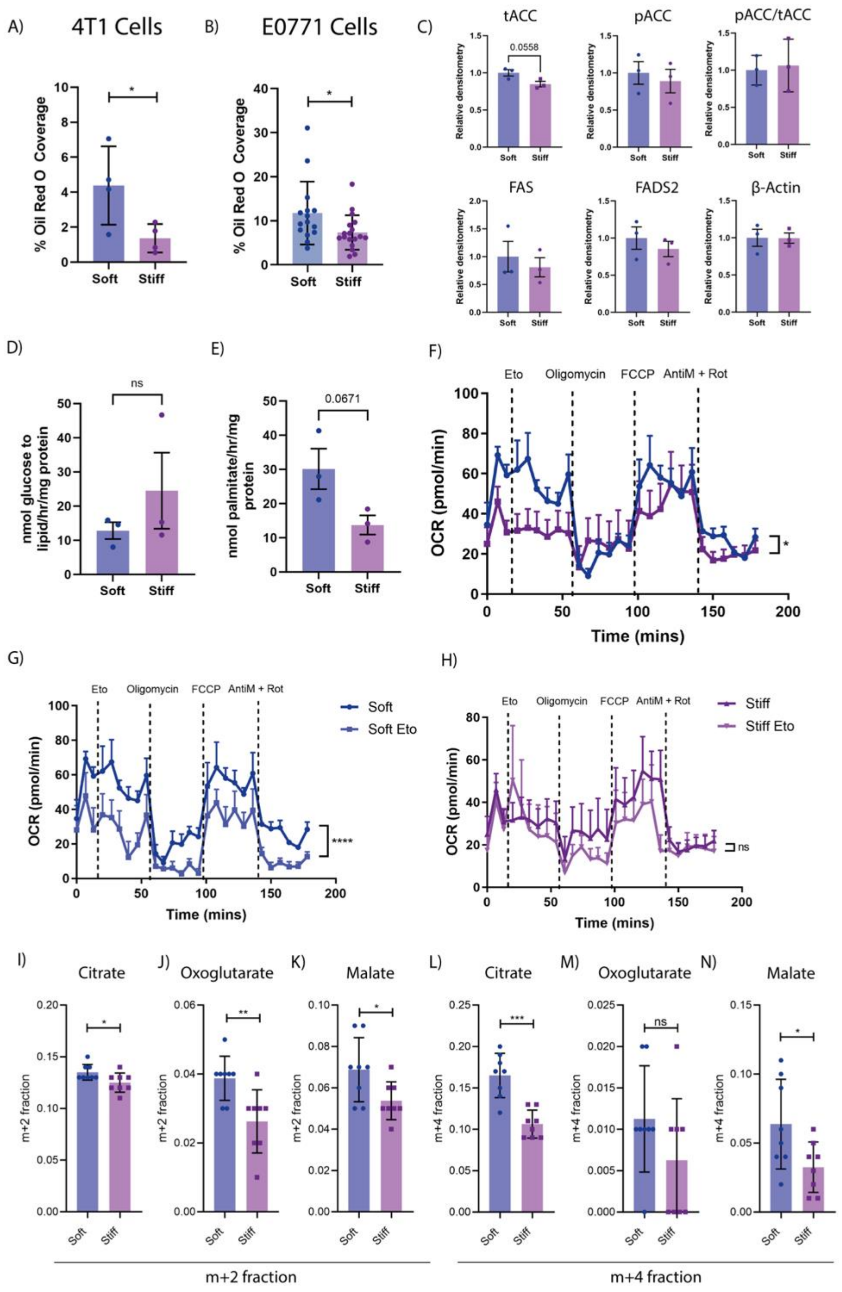

***A**) Quantification of total coverage of Oil Red O staining on 4T1 cells on soft and stiff hydrogels. Graph depicts one biological repeat, representative of n=2 biological repeats. Statistical testing performed using the Mann-Whitney U test. **B**) Quantification of the total Oil Red O staining on E0771 mammary carcinoma cells. Graph depicts one biological repeat, representative of n=2 biological repeats. Statistical testing performed using the Mann-Whitney U test **C**) Quantification of protein densitometry studies, looking at key fatty acid synthesis enzymes (tACC – total Acetyl-CoA carboxylase; pACC – phospho Acetyl-CoA carboxylase; FAS – Fatty Acid Synthase; FADS2 - Fatty acid desaturase 2). n=3 biological repeats. Statistical testing performed using a two-sided unpaired t-test with Welches correction. Radioactive experiments showing incorporation of **D**) de novo synthesized (from ^{14}C Glucose), or **E**) exogenously imported fatty acids (from ^{14}C Palmitate) into the fatty acid lipid reserves. n=3 biological repeats. Statistical testing performed using a two-sided unpaired t-test. **F**) Seahorse*

bioanalyzer plot for the fatty acid stress test on 4T1 cells. Statistical testing performed using a two-way ANOVA. ***G****) and* ***H****) Oxygen consumption traces of stiffness preconditioned 4T1 mammary carcinoma cells with and without fatty acid oxidation inhibitor, Etomoxir. Statistical testing performed using a two-way ANOVA.* ^{13}*C Palmitate metabolomics tracing showing abundances of m+2* ***I****) Citrate,* ***J****) Oxoglutarate,* ***K****) Malate and m+4* ***L****) Citrate,* ***M****) Oxoglutarate and* ***N****) Malate, between the soft and stiff conditions. n=4 biological reps. Statistical testing performed using a two-sided unpaired t-test. * = p<0.05, ** = p<0.01, *** = p<0.001, **** = p<0.0001.*

Figure S6

A)

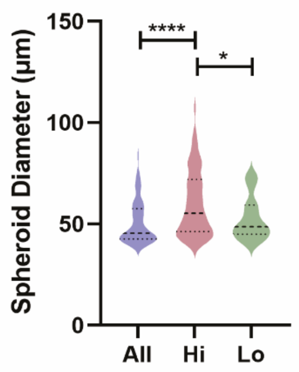

***A**) Quantification of the spheroid diameter at day 5, between BODIPY^{hi}, BODIPY^{lo} and BODIPY^{all} cells. Statistical testing performed using the Mann-Whitney U test. * = $p<0.05$, **** = $p<0.0001$.*

Figure S7

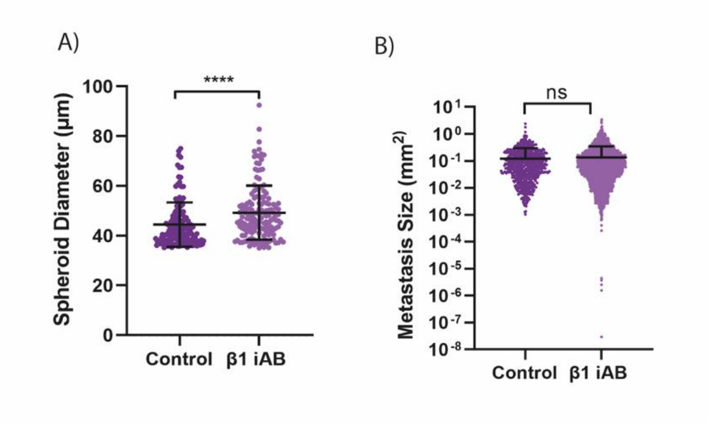

- ***A****) Spheroid diameter measurements at day 5 post embedding with cells primed on stiff substrates, with and without a iAB for Integrin 1β. Graph depicts one biological repeat, representative of n=3 biological repeats.* ***B****) Measurement of metastasis size within the lungs of mice receiving cells primed on stiff substrates, with and without iAB for Integrin 1β. N=7-8 mice per group. Statistical testing performed using a two-sided unpaired t-test throughout. **** = p<0.0001.*

Figure S8

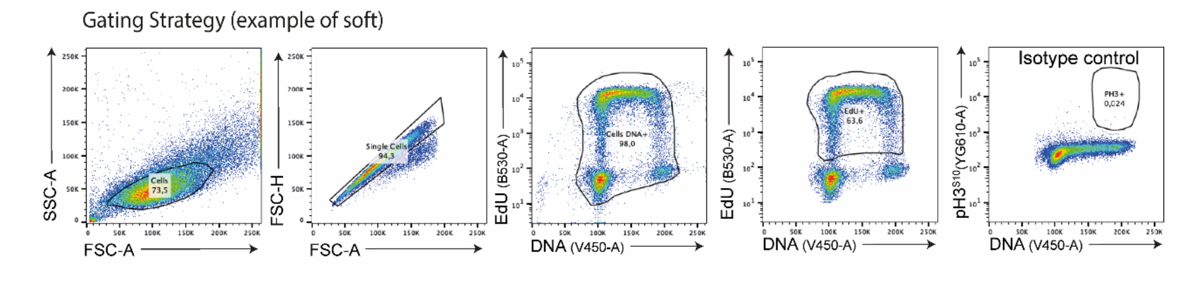

Gating strategy for EdU Proliferation assay. The bulk population was identified from the binate plots of soft conditioned cells, with debris and large cells/doublets excluded using a broad gate on the FSC-H channel. Cells from this gated population were then further analyzed for the DNA and EdU content using the appropriate channels. Further analysis for Histone 3 phosphorylation is also depicted.

Figure S9

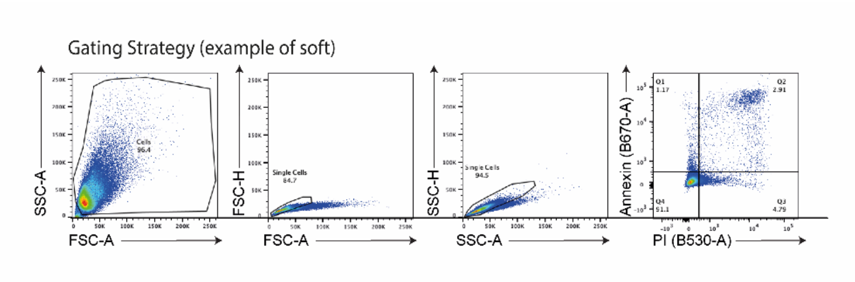

Gating strategy for the Annexin/PI staining post shear stress. The bulk population was identified from the binate plots of soft conditioned cells using the SSC-A vs FSC-A parameters, with debris and large cells/doublets excluded using a broad gate. Cells from this gated population were then further analyzed for Annexin and PI using the B670-A and B530-A parameters, as shown.

Figure S10

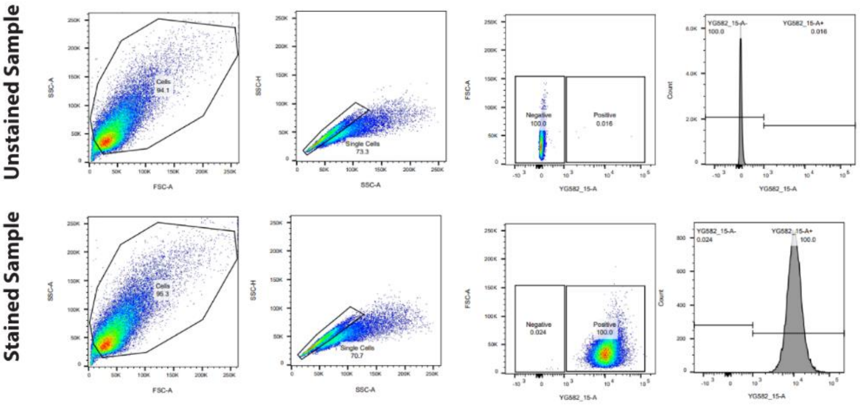

Gating strategy for MitoTracker Orange Flow Cytometry Analysis. The bulk population was identified from the binate plots of unstained cells (top) and for MitoTracker Orange stained cells (bottom, SSC-A vs FSC-A), with debris and large cells/doublets excluded using a broad gate. Cells from this gated population were then further analyzed to ensure single cell isolation and the removal of doublets: SSC-A vs SSC-H. The negative population (YG582-15) from unstained cells was gated and used and applied to determine positive cells from the MitoTracker Orange stained population.

Figure S11

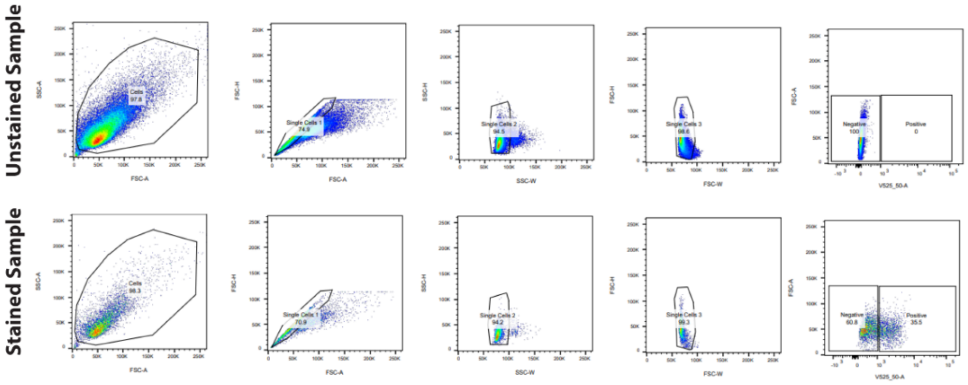

Gating strategy for JC-1 Flow Cytometry Analysis. The bulk population was identified from the binate plots of unstained cells (top) for FCCP treats cells stained with JC-1 (bottom, SSC-A vs FSC-A), with debris and large cells/doublets excluded using a broad gate. Cells from this gated population were then further analyzed to ensure single cell isolation and the removal of doublets using a three step process: FSC-A vs FSC-H, SSC-W vs SSC-H and FSC-W vs FSC-H. The negative population (VS525-) from unstained cells was gated and used and applied to determine positive cells from the FCCP stimulated population of cells stained with JC-1.

Figure S12

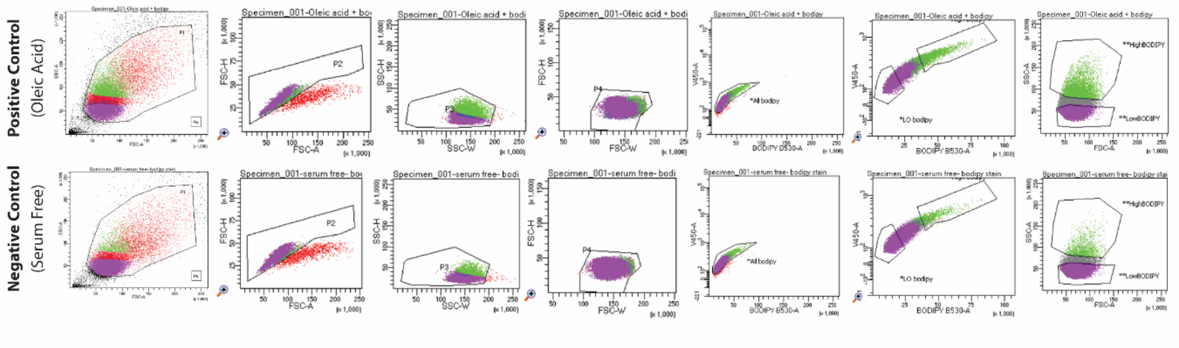

Gating strategy for BODIPY™ Cell Sorting. The bulk population was identified from the binate plots of oleic acid treated BODIPY™ stained cells (top) and serum depleted BODIPY™ stained cells (bottom), with debris and large cells/doublets excluded using a broad gate. Three separate and increasingly stringent preliminary FCS/SSC gates (FSC-A vs FSC-H, SSC-W vs SSC-H and FSC-W vs FSC-H) were applied to the cells to remove all dead cells and doublets from the analysis. Following that, a B530-A (BODIPY) x V450-A (DAPI) density plot was generated, where DAPI positive cells (i.e. dead/dying cells) were excluded. BODIPY^{Hi} cells were collected from the uppermost 10% of the BODIPY stained population whilst the bottom 20% were collected as the BODIPY^{Lo} population. The final FSC-A vs SSC-A flow depicts where the BODIPY^{Hi} and BODIPY^{Lo} populations of cells sit within the side scatter parameter, consistent with increased lipid droplet (cellular granularity).
